# Stabilization and tracking control of underactuated ball and beam system using metaheuristic optimization based TID-F and PIDD^2^–PI control schemes

**DOI:** 10.1371/journal.pone.0298624

**Published:** 2024-02-14

**Authors:** Farhan Zafar, Suheel Abdullah Malik, Tayyab Ali, Amil Daraz, Abdul Rahman Afzal, Farkhunda Bhatti, Irfan Ahmed Khan

**Affiliations:** 1 Department of Electrical and Computer Engineering, Faculty of Engineering and Technology, International Islamic University Islamabad (IIUI), Islamabad, Pakistan; 2 School of Information Science and Engineering, NingboTech University, Ningbo, China; 3 Department of Industrial Engineering, University of Business and Technology (UBT) University, Jeddah, Saudi Arabia; 4 Department of Electronic Engineering, Mehran University of engineering & Technology Jamshoro, Jamshoro, Pakistan; 5 Department of Electrical Engineering, Faculty of Engineering, Universiti Malaya, Kuala Lumpur, Malaysia; National Institute of Technology Silchar, India, INDIA

## Abstract

In this paper, we propose two different control strategies for the position control of the ball of the ball and beam system (BBS). The first control strategy uses the proportional integral derivative-second derivative with a proportional integrator PIDD^2^-PI. The second control strategy uses the tilt integral derivative with filter (TID-F). The designed controllers employ two distinct metaheuristic computation techniques: grey wolf optimization (GWO) and whale optimization algorithm (WOA) for the parameter tuning. We evaluated the dynamic and steady-state performance of the proposed control strategies using four performance indices. In addition, to analyze the robustness of proposed control strategies, a comprehensive comparison has been performed with a variety of controllers, including tilt integral-derivative (TID), fractional order proportional integral derivative (FOPID), integral–proportional derivative (I-PD), proportional integral-derivative (PI-D), and proportional integral proportional derivative (PI-PD). By comparing different test cases, including the variation in the parameters of the BBS with disturbance, we examine step response, set point tracking, disturbance rejection analysis, and robustness of proposed control strategies. The comprehensive comparison of results shows that WOA-PIDD^2^-PI-ISE and GWO-TID-F- ISE perform superior. Moreover, the proposed control strategies yield oscillation-free, stable, and quick response, which confirms the robustness of the proposed control strategies to the disturbance, parameter variation of BBS, and tracking performance. The practical implementation of the proposed controllers can be in the field of under actuated mechanical systems (UMS), robotics and industrial automation. The proposed control strategies are successfully tested in MATLAB simulation.

## Introduction

Underactuated mechanical systems (UMS) have fewer control actuators than their degree of freedom they possess. Modern science and engineering incorporate these systems in various practical and diverse applications. Diverse fields, including robotics, the aeronautical industry, and aerospace, actively use underactuated systems. Furthermore, researchers find these systems of great interest and importance as prototypes for complex nonlinear systems in addition to their practical applications. In recent years, researchers have focused primarily on underactuated systems control design. As the field of UMS continues to emerge, a fundamental challenge arises: the development of a theoretical framework. Through a theoretical perspective, UMS controllability and stabilization is a significant challenge for the control research community. The utilization of underactuated mechanical systems (UMS) in engineering research and education encompasses various applications, with the ball and beam system (BBS) emerging as a particularly renowned and widely-used benchmark. Using a straightforward yet efficient mechanism, it actively illustrates the fundamental principles of control system engineering, encompassing modeling, identification, analysis, and design. The system consists of a ball that travels along a beam and a sensor that measures the position of the ball. An angle adjustment of the beam controls the position of the ball.

Researchers have explored various control strategies, such as Proportional Integral Derivative (PID), Linear Quadratic Regulator (LQR), fuzzy logic, neural networks, adaptive control, and many more upon BBS. This study advanced the development of the PID controller [1] to achieve improved control for the BBS.

Comparisons are made between Fractional Order Proportional Derivative (FOPID) controllers [2–5] and conventional PID controllers, as well as advanced controllers like Linear Quadratic Gaussian (LQG) and H∞. FOPID controllers outperformed all these controllers. A fractional order sliding mode control [6, 7] was proposed to improve the stability and robustness of the system. They controlled a highly nonlinear BBS fractionally in their work. Their conclusion indicated that a FOPID controller offers more freedom than a PID controller.

Regarding variations in system gain, simulations show that fractional order control (FOC) is superior to Integer Order Control (IOC). They employed an optimal control approach [8] to minimize the tracking error of the ball. The results demonstrated that Fuzzy PID [9–11] exhibits superior performance and algorithmic efficiency compared to traditional PID. A more complex problem is addressed by utilizing the sliding mode controller [12]. Simulations and experimental results of BBS verify the effectiveness and demand of the designed control laws.

Metaheuristic approaches are optimization techniques inspired by natural processes such as evolutionary algorithms, swarm intelligence, and simulated annealing. These approaches have proven effective in solving complex optimization problems, including control problems [13]. Metaheuristic algorithms [14] have been applied to the BBS to optimize the control parameters and provide better performance. An algorithm that combines the exploration and exploitation abilities of two or more algorithms to produce an optimal solution is called a hybrid algorithm. Additionally, some studies focused on comparing different metaheuristic algorithms for controlling the BBS. The optimization of the PID controller for the BBS has been done with a Genetic Algorithm (GA) [15, 16]. The results showed that the modified PID outperformed the standard PID search algorithm and other optimization techniques in terms of cost and raised time. A knowledge-based particle swarm optimization (PSO) algorithm [17–19] adaptively controls the BBS. In comparison to H-infinity-based PIDs and Particle Swarm Optimization (PSO)-based I-PDs, they concluded that the Cuckoo Search algorithm CSA-PI-PD [20] serves as a significantly superior controller in terms of closed-loop transient response. The study of the set point tracking response of a BBS involved the employment of a PI-PD controller. Researchers utilized evolutionary computational techniques like genetic algorithms (GA) to determine the optimum parameters of the proposed controller. The assessment of a PI-PD controller included the usage of ITSE, ISE, ITAE, and IAE. Simulation results demonstrate that GA-PI-PD [21] controllers with each performance index are more efficient than SIMC-PID [22] and H-infinity controllers. They investigated the stability of the BBS using a PID controller in their study. Simple internal model control (SIMC) based PID and H-Infinity controller had been proposed and gave satisfactory results. Based on the coefficient diagram method (CDM), their work aims to present a design methodology for a PID controller for an unstable BBS. The study reveals that CDM-PID controllers maintain excellent stability of ball position and exhibit a lower percentage of error compared to ZN-PID controllers [23]. The Simulated Annealing (SA) methodology calibrates the gain coefficients of three control techniques, PID, PIDA, and PI-D to achieve the desired behavior. Analyzing the response of controllers, SA-PIDA demonstrates favorable results, followed by SA-PID and SA-PI*σ*D, respectively [24].

Demonstrating superior effectiveness, the knowledge-based PSO algorithm [25] surpasses BBS, as evidenced by response curves generated through advanced correction, ziegler-nichols, basic PSO, and knowledge-based PSO. In a study, PSO, an artificial bee colony technique (ABC) [26, 27] and a bat algorithm optimization technique (BAO) are among the metaheuristics techniques used to tune PID for cascaded control of BBS. The BAT algorithm has optimized motor position control more efficiently. The PSO algorithm outperformed other algorithms in overall system optimization, considering time response, overshoot, and steady-state error. The Gravitational Search Algorithm (PSO-GSA) and grey wolf optimization (PSO-GWO) [28] hybrid algorithms are applied to tune controller parameters, thereby improving system performance. Compared to other controllers, PSO-GWO controlled the position and angle of the ball more effectively.

In addition to UMS, many types of pendulums are often used in research, such as The Furuta pendulum or rotational inverted pendulum [29] it is a prototype underactuated nonlinear system used as a test bed for various control schemes [30], the inertia-wheel pendulum [31], the acrobat and pendubot [32], and the rotating pendulum [33], the cart pole system [34], the BBS [35], the ball and balancer system [36], the translational oscillator with a rotational actuator TORAsystem [37], and many others. Focusing on control—an observer-based nonlinear robust controller for the BBS has been successfully developed [38]. As the observer output tracks the plant output, the estimation error between them quickly converges to zero. An adaptive control method also provides better control performance in terms of robustness [39, 40]. Controlling the ball position involved implementing an active disturbance rejection control (ADRC) [41], yielding better results compared to PID.

Highly nonlinear or time-varying systems can compromise the performance of PID controllers. The PID controller lacks inherent predictive capability, resulting in erratic behavior when exposed to noise. PID faces difficulties during dead time zones. The complexity and sensitivity of FOPID controllers can lead to a higher implementation cost than traditional PID controllers, along with increased computational resource requirements. In contrast to tuning a simpler controller like a PI or PD, PI-PD controllers can be more complex. PI-PD controllers may encounter challenges when attempting to control systems with strong nonlinearities. PI-PD has limited adaptability to time-varying Systems. Non-standard sensors and unconventional measurement devices may present challenges for PI-PD controllers. In addition to being less robust to parameter changes and more sensitive to noise, PI-D controllers are extremely sensitive to operational noise because of the derivative term amplifying high-frequency components. I-PD controllers may not be suitable for all systems. Handling nonlinearities can be challenging, and adding derivative terms may not be advantageous for systems that undergo minimal dynamic changes or have minimal delays. Non-standard sensors are difficult to integrate with TID controllers. The accuracy of a system model influences the effectiveness of a controller. Research on the control of BBS has made significant progress in improving their performance, particularly in terms of stability and steady-state response. However, there remains a notable research gap in addressing the transient time response of controllers for BBS. The transient time, also known as settling time, is a crucial aspect of system dynamics, representing the duration it takes for the system to reach and stabilize around its desired position after a disturbance or change in set point. The existing literature predominantly focuses on steady-state performance metrics, such as stability and accuracy, while the transient time response is often overlooked. The transient behavior of a control system is essential in applications where rapid and accurate repositioning is crucial, such as in industrial automation, robotics, or precision control systems.

Closing this research gap is essential for achieving optimal performance in ball and beam systems, as an improved transient time response contributes to faster and more accurate system reactions to external disturbances or set point changes. Addressing this gap requires the development and implementation of novel control strategies, tuning methods, or adaptive algorithms that specifically target and optimize the transient response of the system.

There are many challenges while designing the control strategies for the BBS. Like nonlinear dynamics, sensitivity to initial conditions, friction in the system and mechanical constraints, sensors measuring the ball position could introduce noise; the dynamics of the actuators used to tilt the beam could introduce delays or nonlinearities, affecting the system’s response to control inputs. Variations in system parameters (e.g., ball mass, beam length) can occur. Achieving a balance between the speed of response and system stability is crucial.

- Formulation of the objective functions for optimal gains of the controllers.
- Optimization of the objective function with recent metaheuristic algorithms Whale Optimization Algorithm (WOA) and Grey Wolf Optimization (GWO).
- Detailed analysis of stability, the transient, and steady-state performance of the proposed control strategies, WOA/GWO-TID-F and WOA/GWO-PIDD^2^-PI, by employing four distinct performance indices.
- The robustness and disturbance rejection analysis of the proposed control strategies, WOA/GWO-TID-F and WOA/GWO-PIDD^2^-PI is carried out with different test cases subject to change in parameters of the BBS, disturbance, and reference tracking to different ball positions.
- Comprehensive comparison between numerous different control schemes and optimization techniques with the proposed WOA/GWO-TID-F and WOA/GWO-PIDD^2^-PI control strategies.

## Modeling of ball and beam system

Based on the laws of motion and energy, the equation of motion [13, 21–23, 36, 42–44] for the BBS can be derived. As the name implies, the BBS consist of a beam pivoting at one end and a ball rolling along it. Servo motors move the beam up and down so the ball can be positioned on the beam.

**Fig 1:**
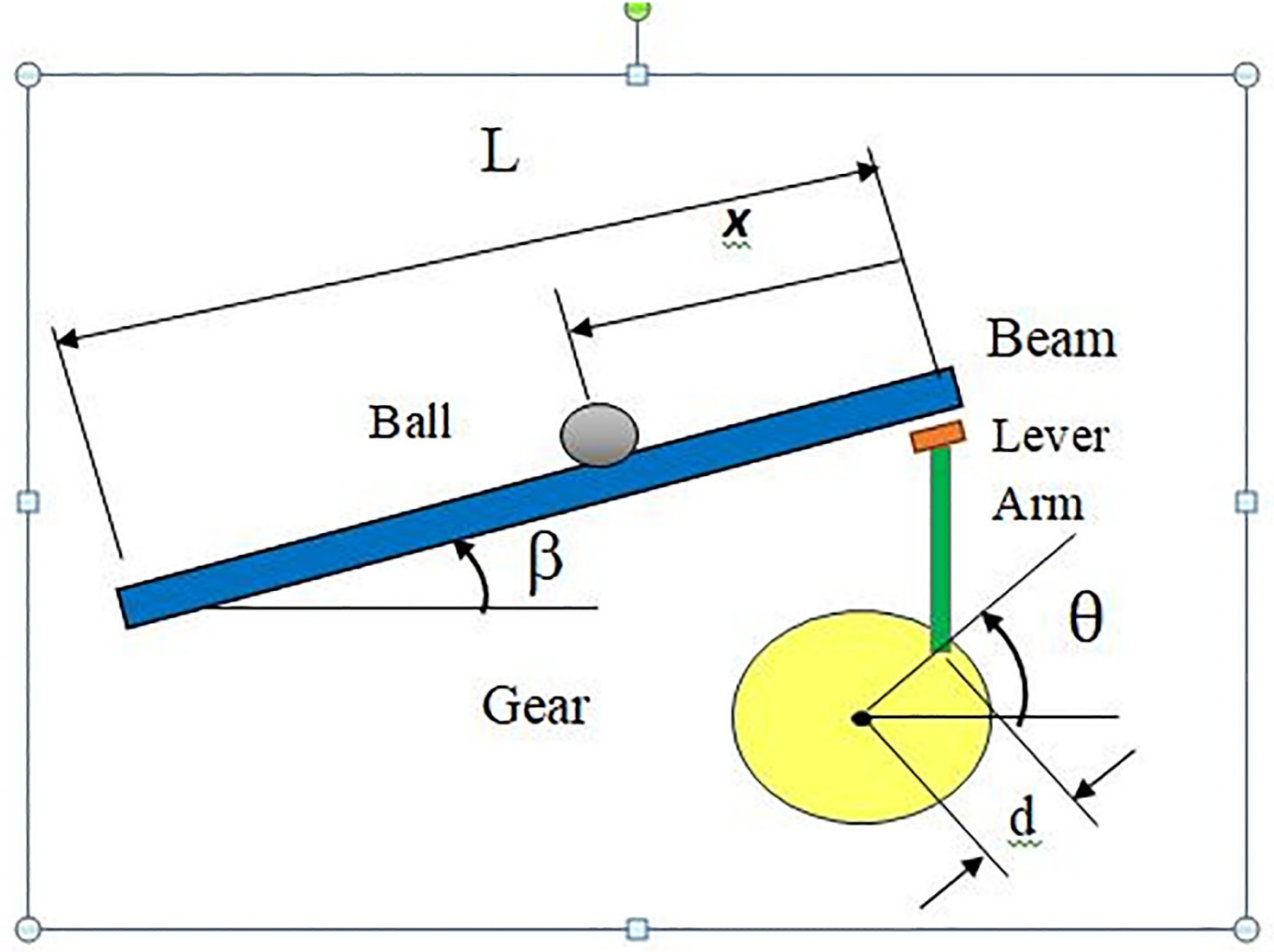
Schematic diagram of BBS.

Fig 1 is showing the schematic diagram of BBS. In which ball has mass m, moment of inertia *J*, radius *r*, and moment of inertia *J*, *θ* (i.e. rotation angle with the wheel) represents the angle between a connecting beam and the horizontal line. The rotation angle of the beam is *α*, the distance between the connecting point of the connecting beam and the gear is *d*, and the length of the crossbar is *L*.

The slope angles *β* and *θ* of the crossbar approximated a proportional relation, and it related as:

$$\begin{array}{c} \beta =\frac{d}{L}\theta . \end{array}$$ (1)

The equation of motion of the BBS system is given as [36].

$$\begin{array}{c} \left(\frac{J}{r^{2}}+m\right)\ddot{x}+mgsin\beta -mx{\dot{\beta }}^{2}=0. \end{array}$$ (2)

When beta is zero the system is in horizontal position and in equilibrium. We can linearize the system with small angle because the value of *sinβ* = *β*

$$\begin{array}{c} \left(\frac{J}{r^{2}}+m\right)\ddot{x}+mg\beta =0. \end{array}$$ (3)

From Eq 1 into Eq 3, we get:

$$\begin{array}{c} \left(\frac{J}{r^{2}}+m\right)\ddot{x}+mg\frac{d}{L}\theta =0. \end{array}$$ (4)

Taking Laplace transformation we get

$$\begin{array}{c} \frac{X(S)}{\theta (S)}=\frac{mgd}{L(\frac{J}{r^{2}}+m)}.\frac{1}{s^{2}}. \end{array}$$ (5)

The moment of inertia is calculated as:

$$\begin{array}{l} J=\frac{2}{5}mr^{2}.\\ \;J=\frac{2}{5}(0.11){\left(0.015\right)}^{2}=9.9e^{-6}kgm^{2} \end{array}$$ (6)

Putting the values of the parameter from Table 1 in Eq 5 we get the final transfer function of ball and beam system

$$\begin{array}{c} G\left(s\right)=\frac{X(S)}{\theta (S)}=\frac{\left(0.11)(-9.8)(0.04\right)}{\left(1\right)\left(\frac{9.9e^{-6}}{0.015^{2}}+0.11\right)}.\frac{1}{s^{2}}=\frac{0.28}{s^{2}}. \end{array}$$ (7)

**Table 1:** Parameters of BBS [20].

Description	Symbol	Values
Mass of the ball	m	0.11 Kg
Gravitational acceleration	G	- 9.8 m/s^2^
Offset of the lever arm	d	0.04 m
Beam’s length	L	1 m
Ball’s moment Inertia of the	J	2/5 mr^2^
Radius of the Ball	r	0.015 m

**Fig 2:**
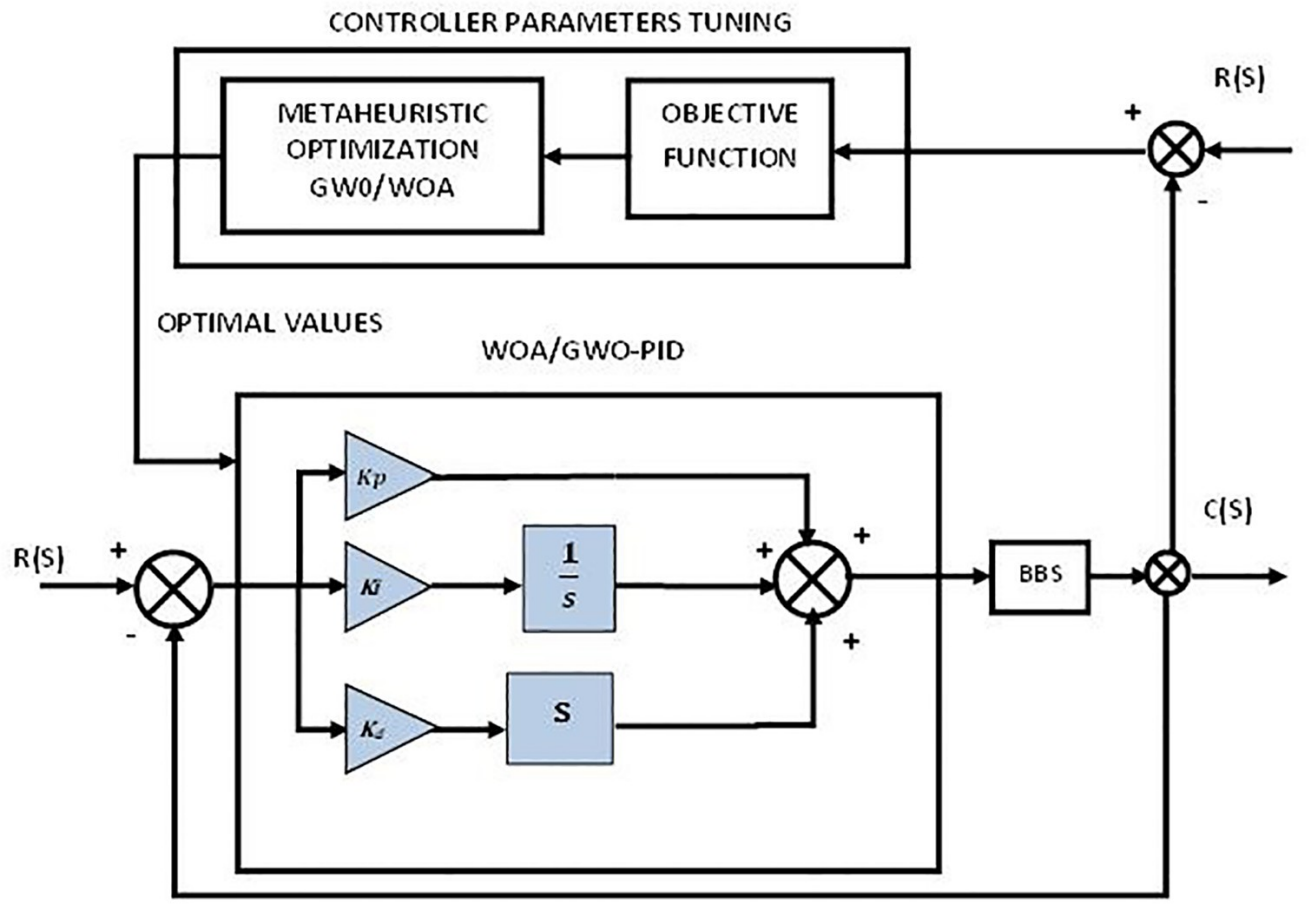
Control scheme of PID.

**Fig 3:**
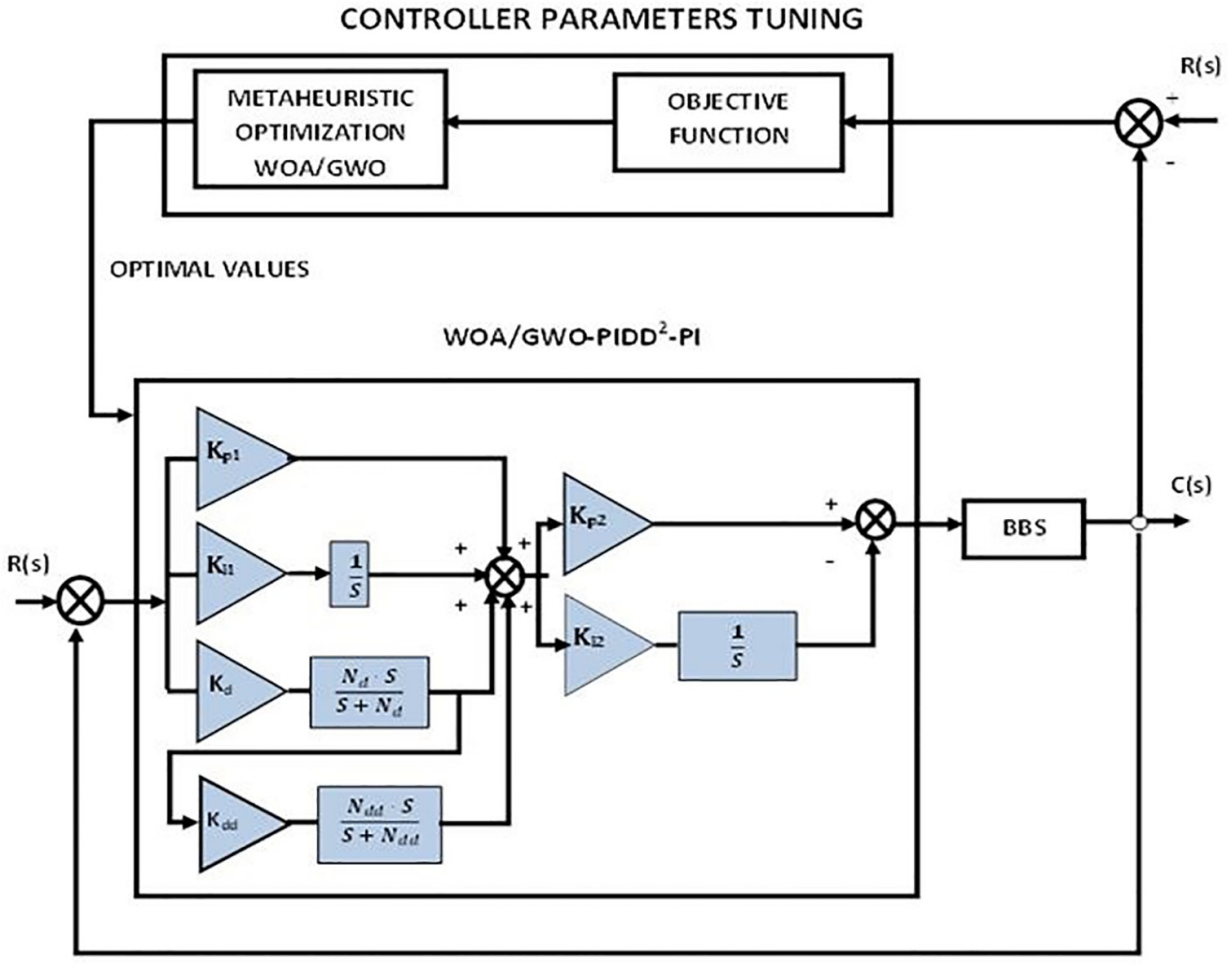
Control scheme of PIDD^2^-PI.

**Fig 4:**
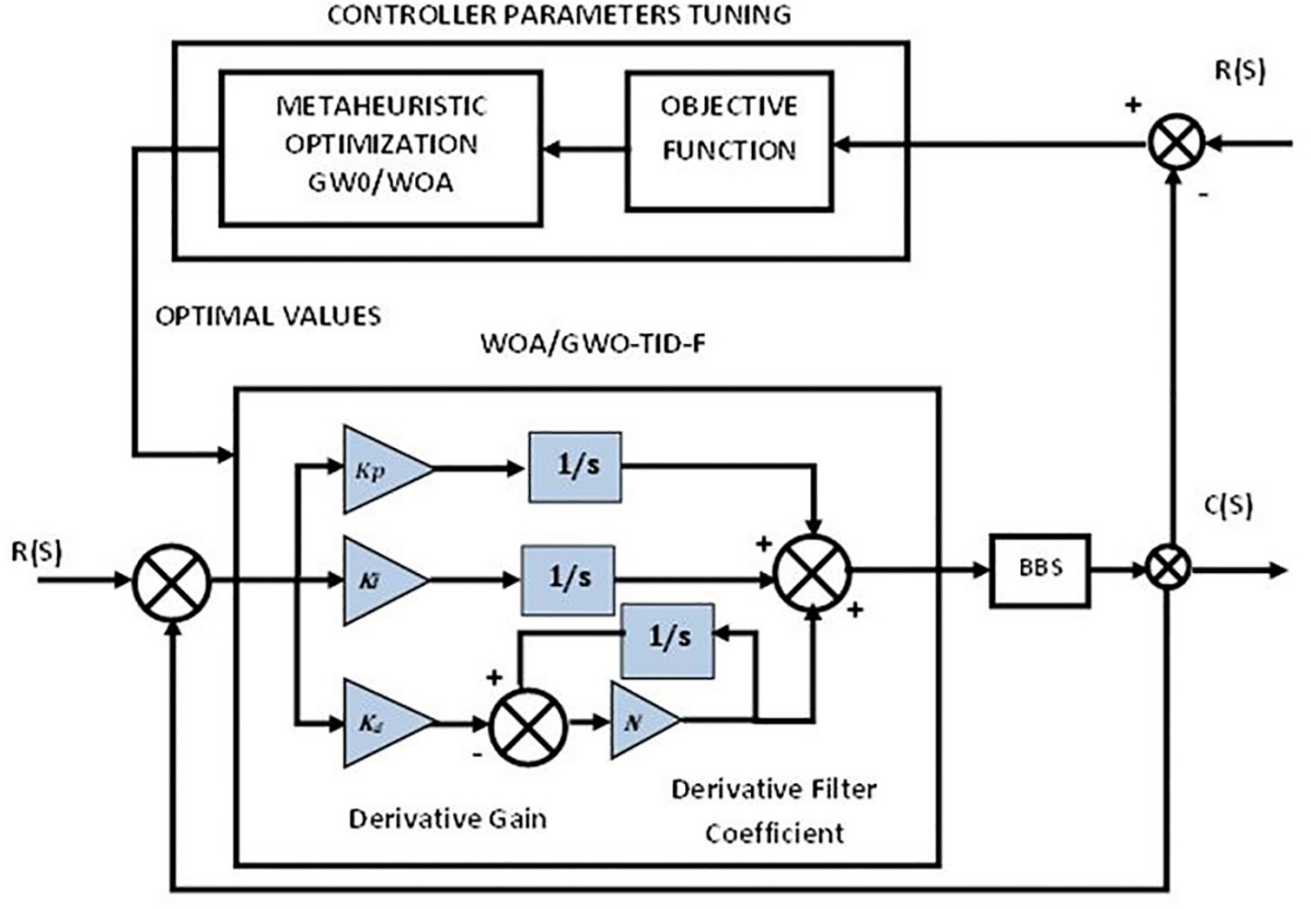
Control scheme of TID-F.

Firstly, the PID controller is tuned using metaheuristic optimization schemes such as GWO and WOA as part of the design methodology then the proportional integral derivative-second derivative with a proportional integrator PIDD^2^-PI, and the tilt integral derivative with filter (TID-F) are two proposed control schemes that are tuned with WOA and GWO are implemented on BBS as shown in Figs 2–4. Details are given below.

The objective is to keep a ball balanced on a beam by controlling the motion of the beam. One of the most used control technique for such systems is PID control.

A PID controller uses feedback to control the system. Using proportional, integral, and derivative terms, the control input is adjusted according to the difference between the desired set point and the actual output. The proportional term adjusts the control input in accordance with the error. As time passes, the integral term adjusts for accumulated errors. By using a derivative term, errors over time can be adjusted. To apply PID control to the BBS, the position of the ball is measured and compared to the desired set point. The error is then fed into the PID controller, which calculates the appropriate control input to adjust the position of the beam. This process is repeated continuously to maintain the ball at the desired position. The closed loop transfer function by using PID is

$$\begin{array}{c} G_{CL}=\frac{Y(S)}{R(S)}=\frac{0.28K_{d}s^{2}+0.28K_{p}s+0.28K_{i}}{s^{3}+0.28K_{d}s^{2}+0.28K_{p}s+0.28K_{i}}. \end{array}$$ (8)

The Proposed controller for the BBS is PIDD^2^ coupled with the PI controller. The controller structure is shown in Fig 3. PIDD^2^ (Proportional Integral Derivative Double Derivative) is a control algorithm used in control systems to regulate a process variable to a desired set point. The PIDD^2^ algorithm is an extension of the classical PID (Proportional Integral Derivative) controller and adds a second derivative term to improve the system’s performance. The PI (Proportional Integral) controller is a basic feedback control system that uses two control actions to regulate a process variable. An integral term represents the cumulative error over time in PIDD^2^ and PI controllers. Proportional terms represent the current error between the set point and the actual process variable. Control performance is improved when the PIDD^2^ and PI controller are combined. The PIDD^2^ algorithm adds a second derivative term to the control signal, which helps to dampen any overshoot or oscillations in the system response. On the other hand, PI controllers adjust the control signal continuously based on the accumulated error of the system to eliminate steady-state errors. The PIDD^2^ coupled with the PI controller working principle can be summarized as follows:

- The process variable is measured and compared to the desired set point.
- Error signals are calculated as differences between set points and process variables.
- A control signal is calculated via the PIDD^2^ algorithm by combining proportional, integral, derivative, and double derivative terms.
- The PI controller adjusts the control signal based on the accumulated error to eliminate steady-state error.
- As a result of the control signal, the actuator adjusts the process variable.

The control loop is repeated to continuously regulate the process variable to the desired set point based on the next measurement of the process variable. Overall, the PIDD^2^ coupled with the PI controller provides a more advanced and robust control system that can handle a wider range of process dynamics and disturbances compared to the classical PID controller. The proposed controller PIDD^2^-PI controller has been optimized with various control optimization algorithms such as GWO and WOA to achieve excellent performance, including a short settling time and rapid rise without overshoot.

There is a similarity between TID’s design [45, 46] and PID’s, though there are some differences; PID is modified by replacing “(1/s)n” with a real number (n) in place of the proportional constant.

TID-F controller is mathematically represented as follows:

$$\begin{array}{c} G_{TID-F}=\frac{K_{T}}{s^{\frac{1}{n}}}+\frac{K_{I}}{s}+K_{D}\left(\frac{N_{s}}{s+N}\right). \end{array}$$ (9)

K_T_, K_D_, and K_I_ represent proportional/tilt, derivative and integral constants on controllers, respectively. The TID controller can be characterized as a combination of fractional order (FO) and integer controllers. TID has an advantage over FO and integer controllers. This method quickly eliminates disturbances between integers and FOs. Where derivative filter coefficient is defined by the parameter N and the Fig 4 shows the TID-F controller’s structure.

By measuring the difference between the desired output and the actual output of a control system, performance indices, or error criteria, are used to evaluate the performance of control systems. There are several types of performance indices, including the Integral of Absolute value of Error (IAE), Integral of Time multiplied by Absolute value of Error (ITAE), Integral of Squared Error (ISE), and Integral of Time multiplied by squared value of Error (ITSE) [36].

IAE measures the cumulative absolute error over time, giving more weight to larger errors. ITAE is similar to IAE but gives more weight to errors that occur earlier in the system’s response. ISE measures the cumulative squared error over time, giving more weight to larger errors. ITSE is similar to ISE but gives more weight to errors that occur earlier in the system’s response. Over some defined period of time T, these performance indices are as follows:

$$\begin{array}{c} ITAE=\int_{0}^{T}t\left|e\left(t\right)\right|\;dt. \end{array}$$ (10)

$$\begin{array}{c} ITSE=\int_{0}^{T}te^{2}\left(t\right)\;dt. \end{array}$$ (11)

$$\begin{array}{c} ISE=\int_{0}^{T}e^{2}\left(t\right)\;dt. \end{array}$$ (12)

$$\begin{array}{c} IAE=\int_{0}^{T}\left|e\left(t\right)\right|\;dt. \end{array}$$ (13)

## Metaheuristic computation for controller tuning

Metaheuristic computational techniques are important for solving optimization problems because they provide efficient and effective solutions when exact methods are impractical or too time-consuming. These techniques can be used in situations where the problem is constantly changing or dynamic, requiring quick adjustments to the solution approach. These are an essential tool for solving optimization problems in a timely and effective manner. Two recently introduced metaheuristic optimization algorithms have been used in this paper. Controllers are tuned using metaheuristic techniques to get the optimum results, and details are given in this section.

### Grey Wolf Optimization algorithm (GWO)

**Fig 5:**
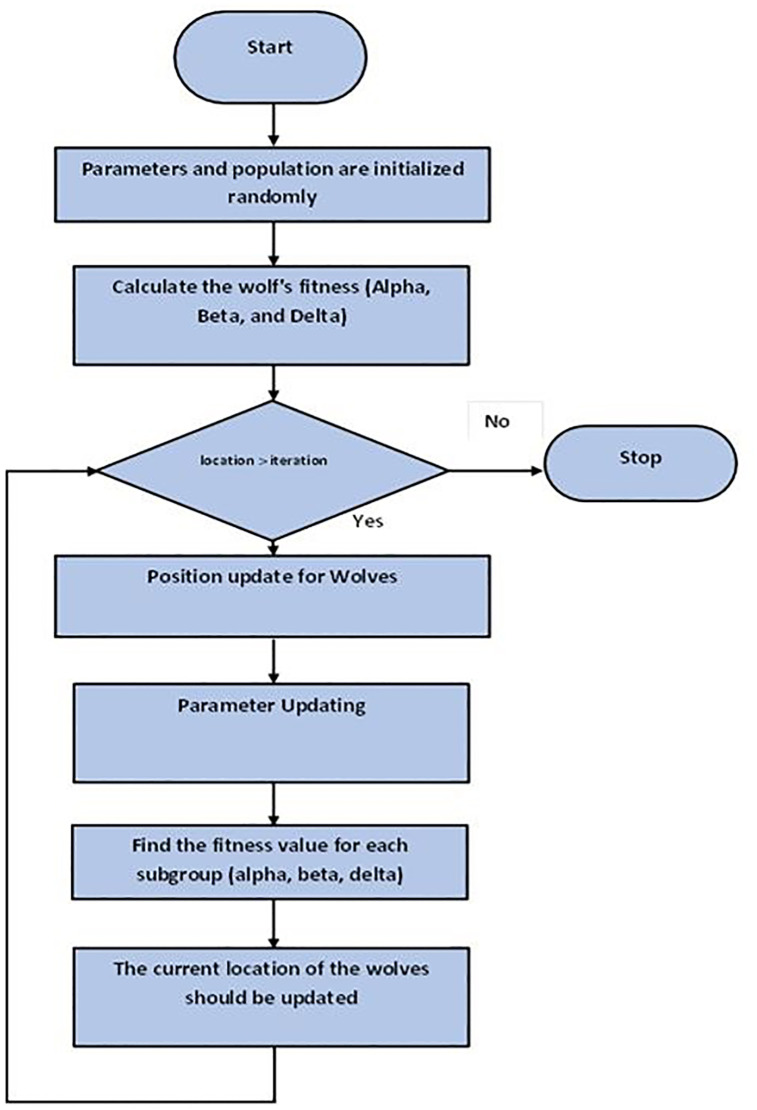
Flow chart of GWO.

GWOs [47] are motivated by grey wolves’ intelligent hunting tactics and social structure. It is common for grey wolves to live in groups of 5 to 12 individuals. Its primary goal is to develop the candidate solution during each iteration, which makes GWO different from other metaheuristic optimization algorithms. In other words, GWO imitates gray wolf hunting behavior, which involves locating and attacking prey. Grey wolves undergo the following stages of hunting as outlined by [48–53]. The process of following, pursuing, and moving forward with the prey continually following, encircling, and harassing the prey. Targeting prey with an attack. Using the GWO algorithm to tune PID controller parameters has exploded in control engineering publications. In recent years, grey wolf optimizer (GWO) has become one of the most popular metaheuristic swarm intelligence methods. The initial search for this technique does not require any derivation information, which makes it more efficient than other swarm intelligence techniques. GWO is organized into four groups based upon the various roles that the wolves play in progressing the hunting process. It has been determined that alpha is the most successful hunting strategy out of the four, with beta, delta, and omega representing the others. In nature, gray wolves are divided into four groups based on their dominance structures. The creators of this algorithm carried out a thorough trial and discovered that taking. A grey wolf population is established as a random population in the GWO search procedure, like previous swarm intelligence algorithms. The four wolf groups and their positions are then established, and the distances to the intended prey are calculated. An update is made on each wolf as it symbolizes a potential solution during the search process. To prevent the local optima from stagnating, keep up the exploration and exploitation. Its mathematical model differs from that of other population-based algorithms in that it determines the global optimum by calculating the value of the global average. To mimic gray wolves hunting and encircling their prey in the wild, it moves a solution around another in an n-dimensional space. GWO only requires a position vector, thereby requiring less memory. Additionally, GWO only retains three best solutions. One of the SI algorithms with the fastest growth is GWO. The success of the GWO algorithm inspires other academics to use the approach to address various optimization issues. The flow chart for GWO is shown in Fig 5.

### Whale Optimization Algorithm (WOA)

Humpback whales’ natural hunting behavior is used to design the whale optimization algorithm WOA [54–58], which involves two main behaviors: searching and encircling prey. The algorithm uses these two behaviors to iteratively improve the fitness of the solution set. In the search phase, the whales move randomly toward the prey, while in the encircling phase, they surround the prey to trap it. By combining these two behaviors, the WOA algorithm forms a multi-objective optimization problem, with the goal of minimizing fitness.

#### General Structure of WOA

It only requires one parameter (time interval) to be adjusted in the algorithm, using a small number of control parameters. It is based on the assumption that a population of humpback whales searches for food in a multidimensional space, in which individuals’ positions is represented by decision variables, and the distance between individuals and food is reflected in objective costs. There are three operational phases involved in whale action during its time-dependent location: shrinking encircling the prey, bubble-net at-tacks, and searching for prey. These operational processes are described and mathematically expressed in the following subsections.

#### Encircling prey

Detecting prey and surrounding it is one of the skills of humpback whales. WOA assumes that the current most appropriate candidate solution represents the target prey or is near the optimal design since the exact position of the optimal design in the search space is unknown beforehand. While the algorithm seeks to identify the most efficient search agent, the remaining search agents adjust their positions around the most efficient search agent. The following equations are used to describe this behavior mathematically:

$$\begin{array}{c} \vec{D}=\left|\vec{C}.\vec{X^{*}}\left(t\right)-\vec{X}\left(t\right)\right|. \end{array}$$ (14)

$$\begin{array}{c} \vec{X}\left(t+1\right)=\vec{X^{*}}\left(t\right)-\vec{A}.\vec{D}. \end{array}$$ (15)

$$\begin{array}{c} \vec{A}=2\vec{a}.\vec{r}-\vec{a}. \end{array}$$ (16)

$$\begin{array}{c} \vec{C}=2.\vec{r}. \end{array}$$ (17)

Where *D* is the distance vector, that specifies the difference between the current position *X*(*t*) and the target position. *C* is the coefficient vector used to update the encircling mechanism in the algorithm X*(*t*) signify the position vector of best solution. *X*(*t* + 1) denotes the updated position vector for the next and A is the coefficient vector used to update the position which depends on two variables *r* and *a* where *a* is a parameter that controls the spiral updating mechanism. *r* is a random vector ranging between 0 and 1, used to introduce randomness in the algorithm.

#### Bubble-net attacks

Bubble-net attacks are cooperative feeding behavior displayed by groups of humpback whales. During a bubble-net attack, a group of whales works together to create a ring of bubbles around a school of fish, which encloses the prey and makes it difficult for them to escape. The whales then take turns swimming through the ring with their mouths open, scooping up large amounts of fish in each pass.

#### Searching for prey

**Fig 6:**
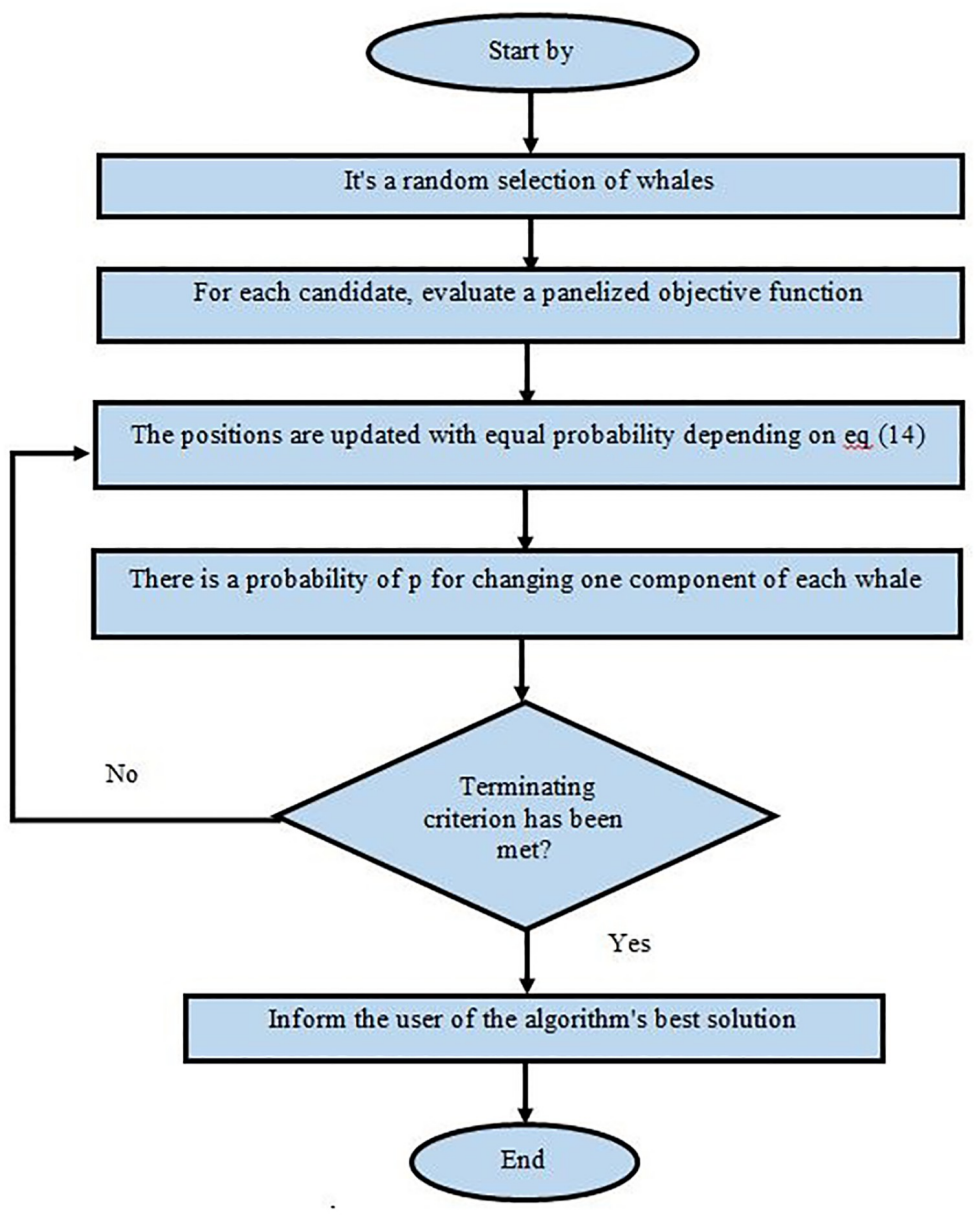
Flow chart of WOA.

Hunting requires exploring the search space to find the optimal solution. In WOA, the whales represent potential solutions, and they move randomly in the search space to search for the prey, which is the optimal solution. Set of mathematical equations guide whale movements in a manner similar to how humpback whales hunt bubble-nets. A balanced exploration-exploitation phase balance is central to the algorithm’s goal of convergent to a global optimum. The flow chart for WOA is shown in Fig 6.

## Simulation results and discussion

**Fig 7:**
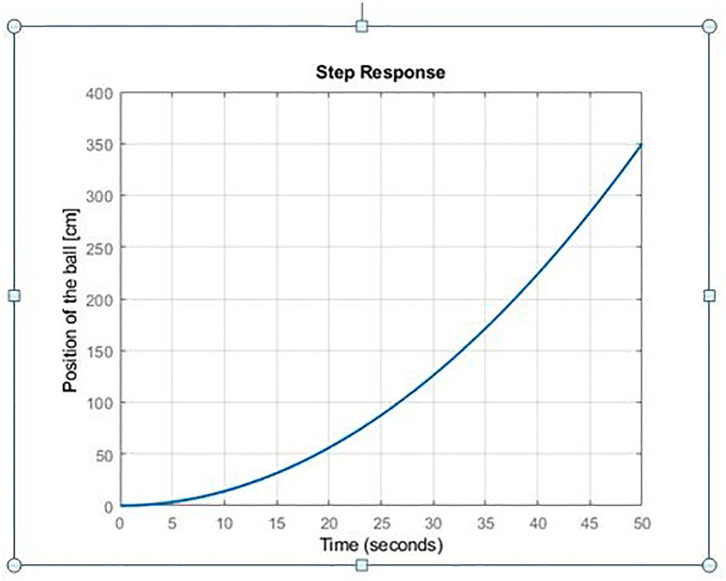
Step response of open loop ball & beam system.

In this section, GWO/WOA-TID-F and GWO/WOA-PIDD^2^-PI controllers, which are designed to provide control of the Ball & Beam system with four performance indices, ITAE, ISE, IAE and ITSE are simulated. For simulations, MATLAB/SIMULLINK software is employed. The open loop response of a ball and beam system is examined first. The system’s open-loop step response is shown in Fig 7. A growing response indicates that the system exhibits an unstable open loop response.

Now feedback control is applied using GWO/WOA-TID-F & GWO/WOA-PIDD^2^-PI. The performance of controllers has been evaluated using three different parameters of transient response, including rise time, overshoot and settling time. The optimum gains of TID-F tuned by GWO & WOA are shown in Table 2.

**Table 2:** Parameters for GWO-TID-F & WOA-TID-F.

Controller	Index	Control Parameters				
		KT	Ki	Kd	n	N
**GWO-TID-F**	**ITAE**	947.276	1.0392	235.2128	62.23	290.434
	**ITSE**	947.033	11.2203	787.232	44.827	959.797
	**ISE**	56.112	15.4257	999.7182	79.154	963.936
	**IAE**	884.515	0.0331	773.6802	24.37	993.127
**WOA-TID-F**	**ITAE**	685.995	3.8830	980.0656	0.0625	648.5159
	**ITSE**	427.12	26.22	987.2320	644.72	454.78
	**ISE**	0.5918	12981	1001.535	871.88	583.454
	**IAE**	484.44	51.0355	983.71	586.04	503.177

**Fig 8:**
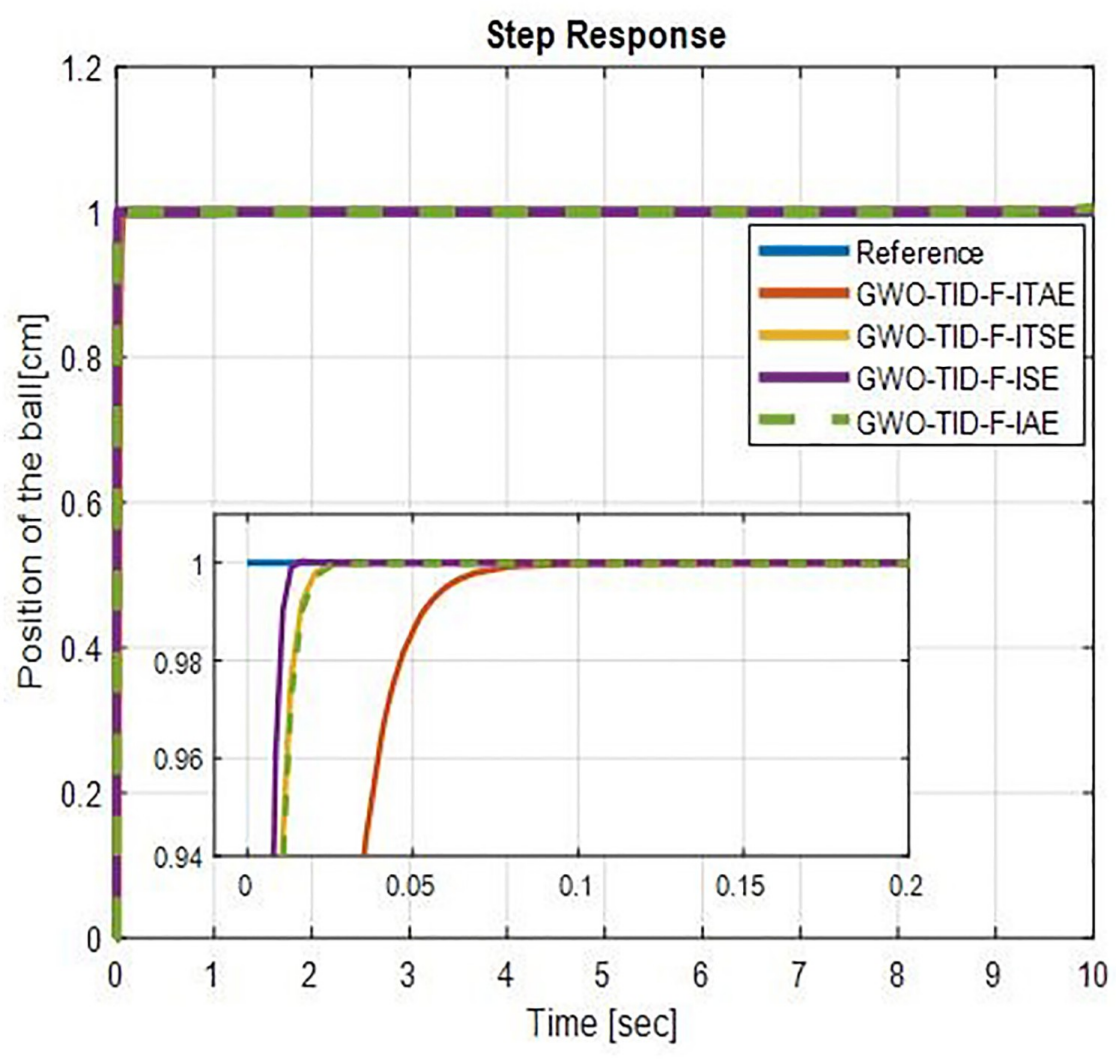
Step response of BBS with GWO-TID-F controller.

**Fig 9:**
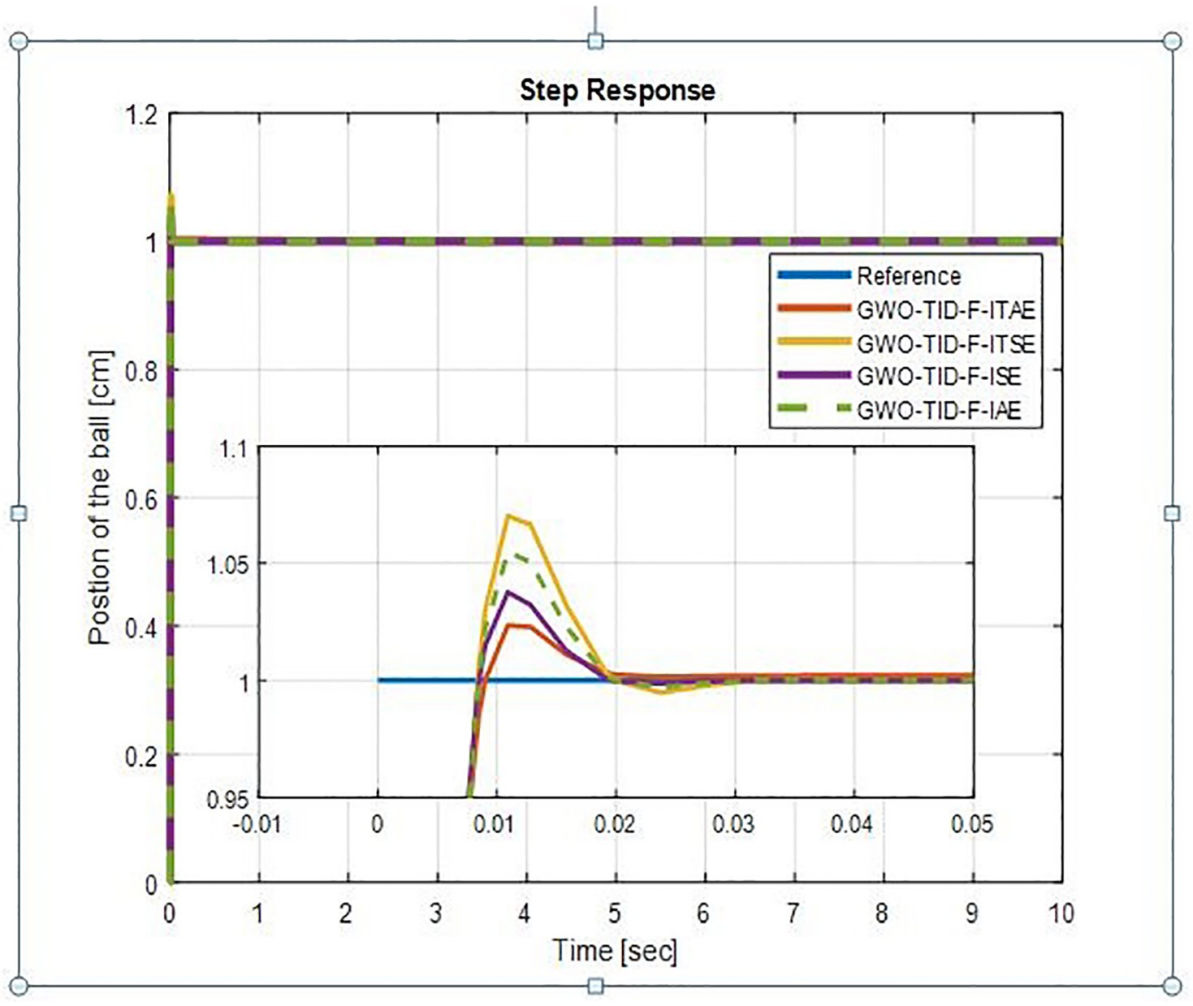
Step response of BBS with WOA-TID_F controller.

It is evident from Fig 8 GWO-TID-F gives excellent performance in terms of rise time, settling time and overshoot. The proposed controller has zero overshoot on all four performance indices. GWO-TID_F with ISE has a lower rise time of 0.0058 seconds and a very short settling time of 0.01 seconds. The 2nd most suitable is GWO-TID_F with ITSE whose rise time is 0.0079 sec and settling time is 0.0141 seconds with zero overshoot. GWO-TID_F with other performance indexes ITAE and IAE also gives an excellent response with a very slight increase in rise and settling time with zero overshoot as com-pared with ITSE and IAE. Step response of WOA-TID-F controller is shown in Fig 9. We can see the rise time and settling time are very short with small overshoots with all indices. The step response performances of BBS with GWO-TID-F & WOA-TID_F Controller are presented in Table 3.

**Table 3:** Step response of GWO-TID-F & WOA-TID-F.

Controller	Index	Performance Parameters		
		Rise Time	Settling Time	% Overshoot
**GWO-TID-F**	**ITAE**	0.0263	0.047	0
	**ITSE**	0.0079	0.0141	0
	**ISE**	0.0057	0.0099	0
	**IAE**	0.0083	0.0158	0
**WOA-TID-F**	**ITAE**	0.0059	0.0135	2.3271
	**ITSE**	0.0077	0.0170	6.9145
	**ISE**	0.0063	0.0146	3.7112
	**IAE**	0.0058	0.0162	5.4165

In comparison to WOA, the TID-F tuned better with GWO. From Table 3 it can be clearly seen that GWO-TID-F provides excellent results. Therefore it can be inferred that ISE index with the proposed controller can tackle BBS with improved dynamic and steady state performance. The other proposed controllers are WOA-PIDD^2^-PI & GWO-PIDD^2^-PI whose optimum control gains are shown in Table 4.

**Table 4:** Control parameters for WOA-PIDD^2^-PI & GWO-PIDD^2^-PI.

Controller	Index	Control Parameters							
		Kp1/KT	Ki	Kd	Kp2	Kdd	Ki2	Nd	Ndd
**WOA-PIDD^2^-PI**	**ITAE**	0.00899	24.2347	4000.76	0.6767	0.3494	0.001467	931.54	9955
	**ITSE**	923.3007	93.3318	986.2217	0.6123	0.3412	0.00156	945.3298	989
	**ISE**	1.786	3.87	1986.56	9.66618	0.21876	9.143e-5	9979.5	9999.6
	**IAE**	0.000233	0.2117	2.4332	96.9861	0.0487	32.3216	685.5898	0.0419
**GWO-PIDD^2^-PI**	**ITAE**	1983.0525	906.5582	6.9653	9.5663	0.210386	0.009933	9697.6	989.5
	**ITSE**	66.4924	75.8543	999.8847	2.1123	0.01187	0.001511	9934	255
	**ISE**	999.8381	78.6452	73.6862	5.9813	0.01185	0.00101	434	752.5
	**IAE**	956.0869	842.6793	582.126	7.5663	0.210386	0.00992	19697.6	989.5

**Fig 10:**
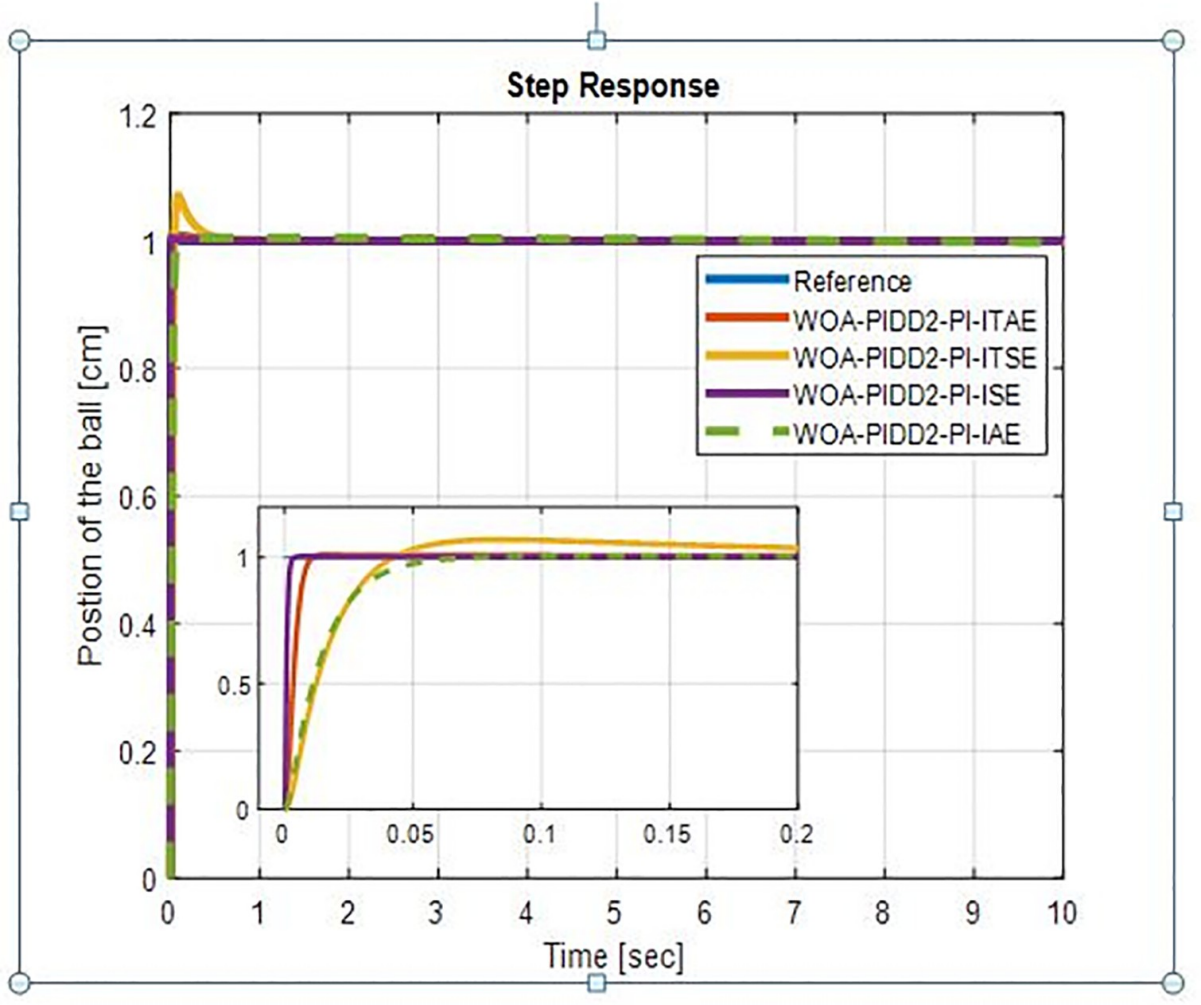
Step response of BBS with WOA-PIDD^2^-PI.

The step response of WOA-PIDD^2^-PI controller is shown in Fig 10. WOA-PIDD^2^-PI with ISE has excellent results as compared to others.

**Fig 11:**
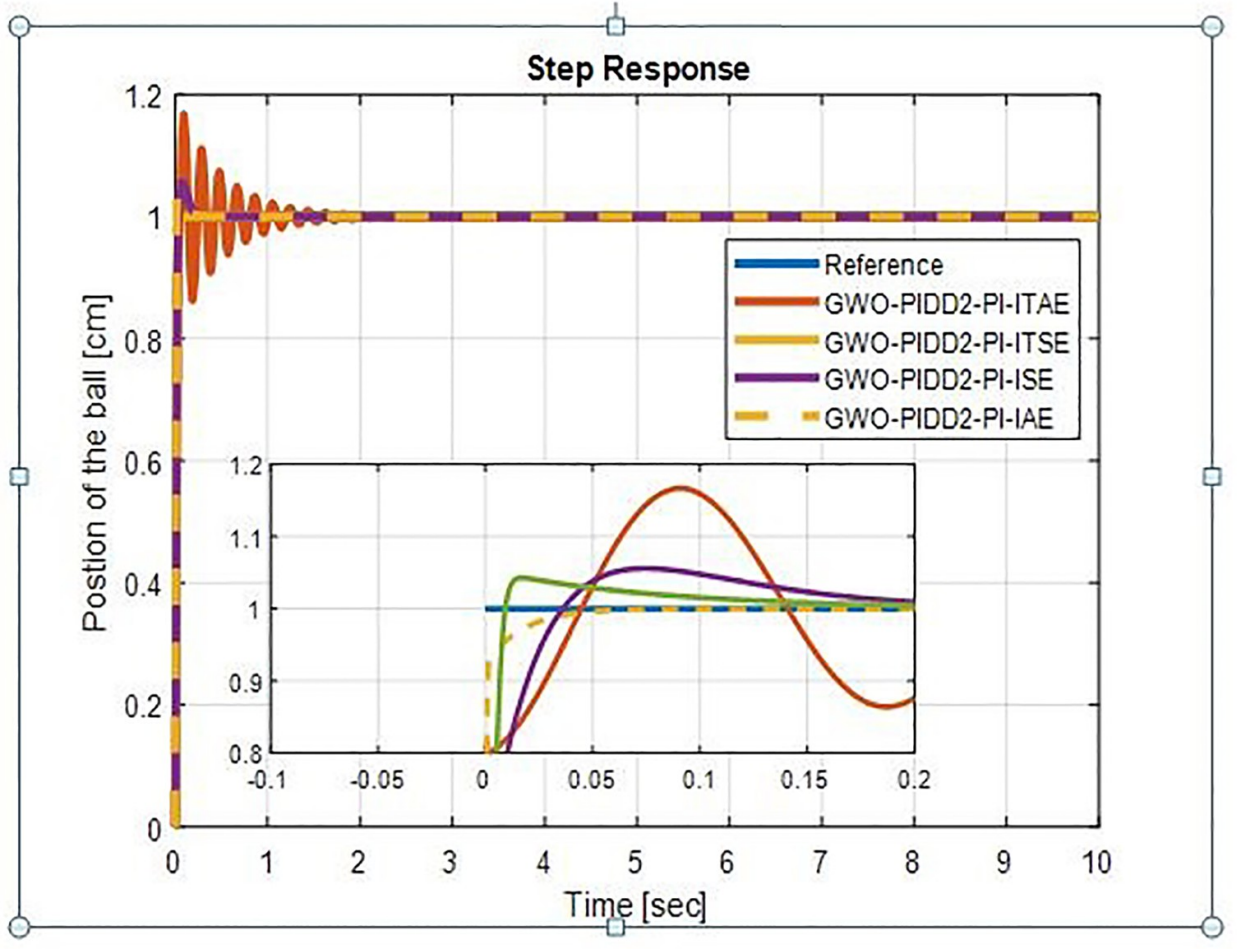
Step response of BBS with GWO-PIDD^2^-PI.

The step response of BBS for GWO-PIDD^2^-PI is shown in Fig 11. The GWO-PIDD^2^-PI with ITSE rise time is 9.8958e-04 sec and settling time is 0.0232 sec with very small percentage overshoot as 0.03% and GWO-PIDD^2^-PI with ITAE gives high overshoots. The step response characteristics of WOA-PIDD^2^-PI & GWO-PIDD^2^-PI controller with four indices are shown in Table 5.

**Table 5:** Step response characteristics of WOA-PIDD^2^-PI.

Controller	Index	Performance Parameters		
		Rise Time	Settling time	% Overshoot
**WOA_PIDD^2^-PI**	**ITAE**	0.0058	0.0093	1.1509
	**ITSE**	0.0268	0.3061	7.1614
	**ISE**	0.0014	0.0025	0
	**IAE**	0.0295	0.0513	0
**GWO-PIDD^2^-PI**	**ITAE**	0.0278	1.0625	16.6635
	**ITSE**	9.8958e-04	0.0232	0.03
	**ISE**	0.0188	0.1588	5.6366
	**IAE**	0.0056	0.0796	4.2839

From Table 5 we see that WOA-PIDD^2^-PI is performing excellent with ISE & IAE WOA-PIDD^2^-PI-ISE rise time is 0.0014 sec & settling time is 0.0025 sec where WOA-PIDD^2^-PI-IAE rise time is 0.0295 sec and settling time is 0.0513 sec. so we choose WOA-PIDD^2^-PI-ISE as a proposed controller.

We implement variety of controllers on BBS for the sake of validity and comparison with the two proposed controllers. The controller which are observed are Tilt- Integral-Derivative (TID), Fractional Order Proportional Integral Derivative (FOPID), Integral –Proportional Derivative (I-PD), Proportional Integral-Derivative (PI-D), Proportional Integral –Proportional Derivative PI-PD tuned with WOA and GWO with four performance indices and choose optimal results for comparison with the proposed controllers. The control gains are shown in Table 6.

**Table 6:** Control parameters for optimal controllers.

Controller	Control Parameters						
	Kp1/KT	Ki	Kd	Kp2	n	μ	l
**GWO_PI-PD-ITAE**	651.701	835.325	135.2707	104.937	-	-	-
**GWO_PI-PD-IAE**	681.060	931.458	97.1397	143.753	-	-	-
**GWO-I-PD-IAE**	684.907	321.010	39.4511	-	-	-	-
**GWO-I-PD-ITAE**	951.410	402.522	59.9527	-	-	-	-
**GWO_PI-D-ITSE**	992.982	78.1452	74.1862	-	-	-	-
**WOA-PI-PD-ISE**	91.5534	2.8842	53.8563	0.9766	-	-	-
**WOA-PID-ITAE**	100.000	0.3465	22.8053	-	-	-	-
**WOA-TID-ITAE**	61.5225	0.0239	45.9257	-	3	-	-
**WOA-FOPID-ITSE**	1.8392	19.4687	100.000	-	-	0.5	0.5

**Fig 12:**
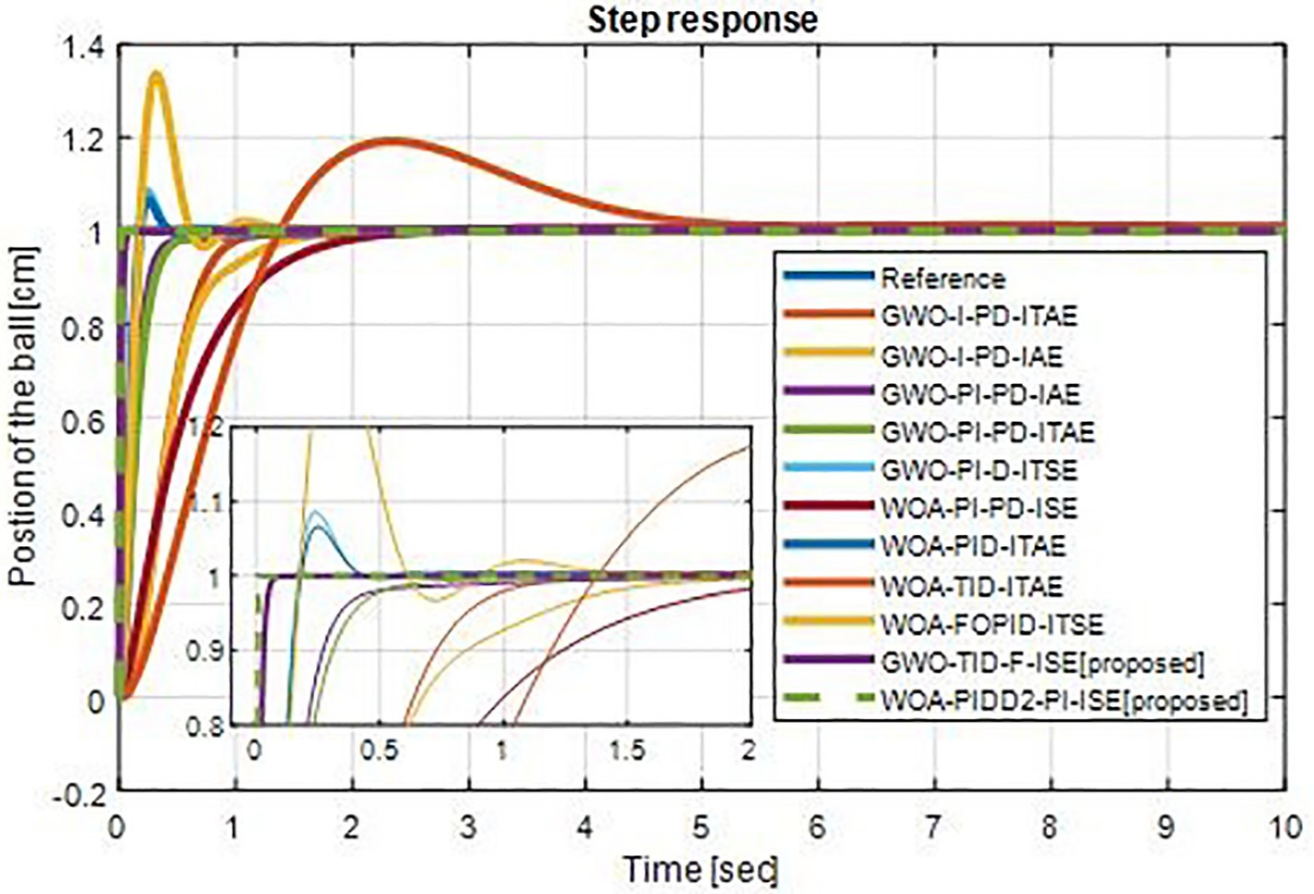
Step response ball & beam system with optimum controllers.

**Fig 13:**
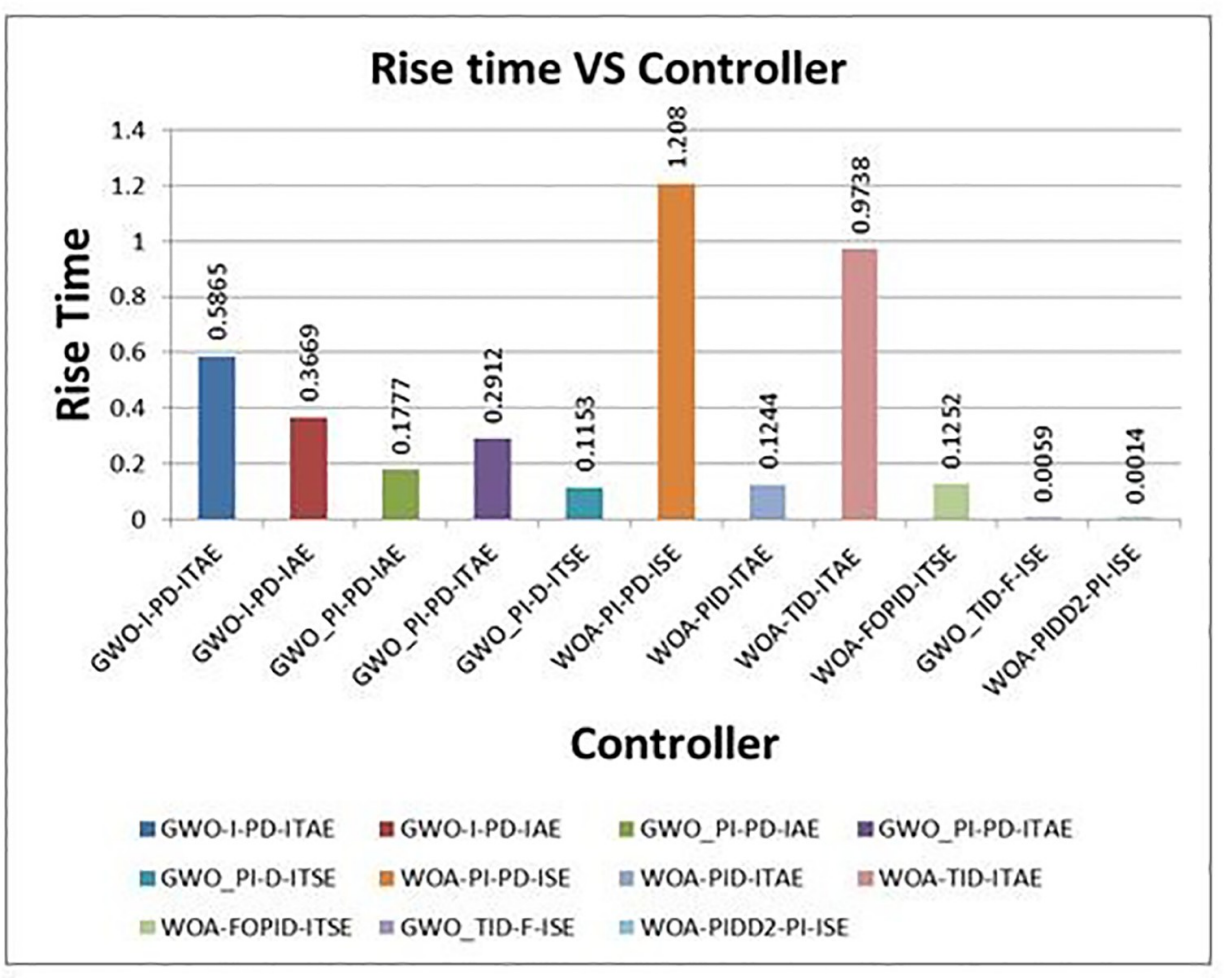
Graphical representation of rise time vs. controllers.

**Fig 14:**
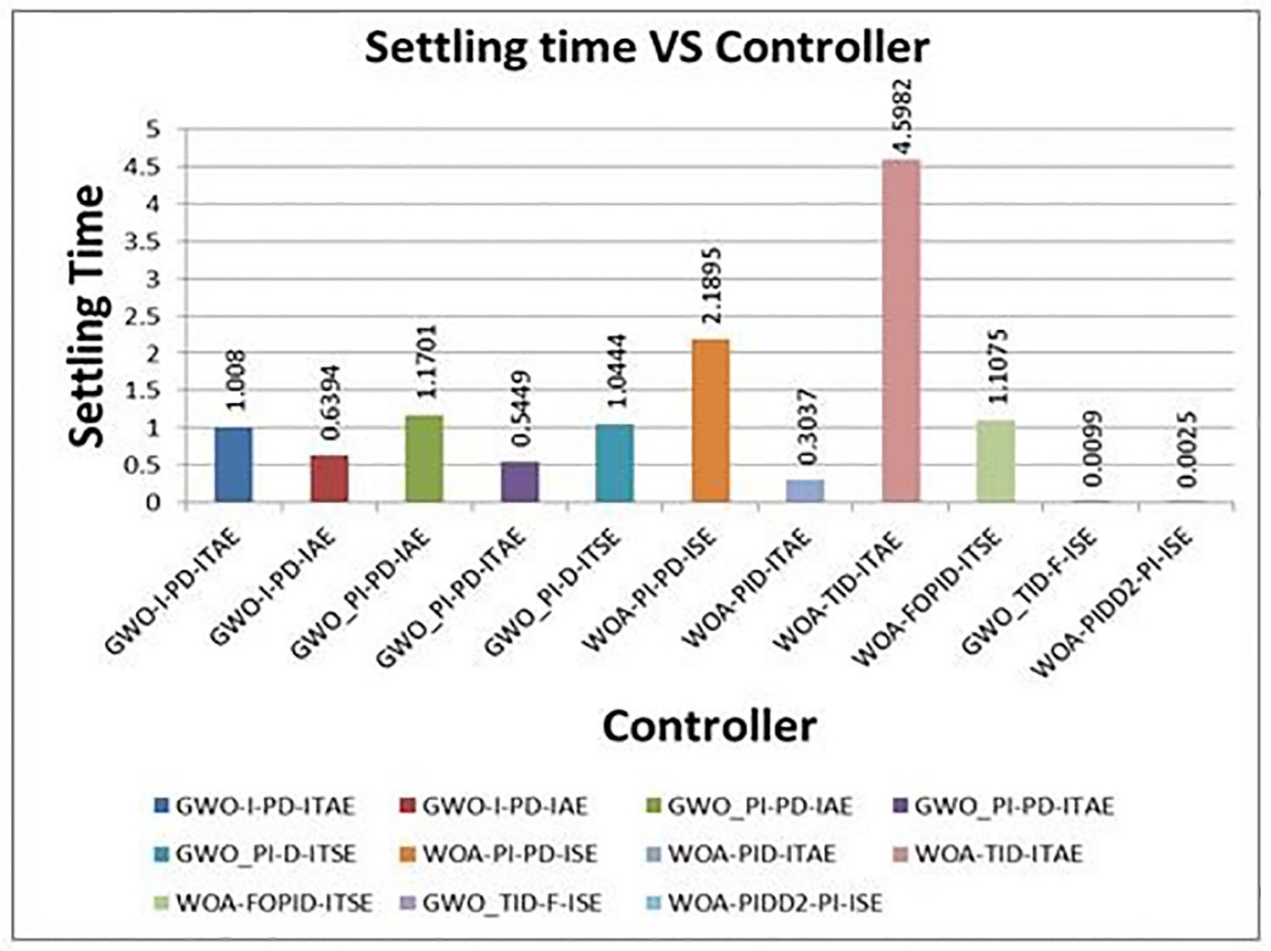
Graphical representation of settling time vs. controllers.

**Fig 15:**
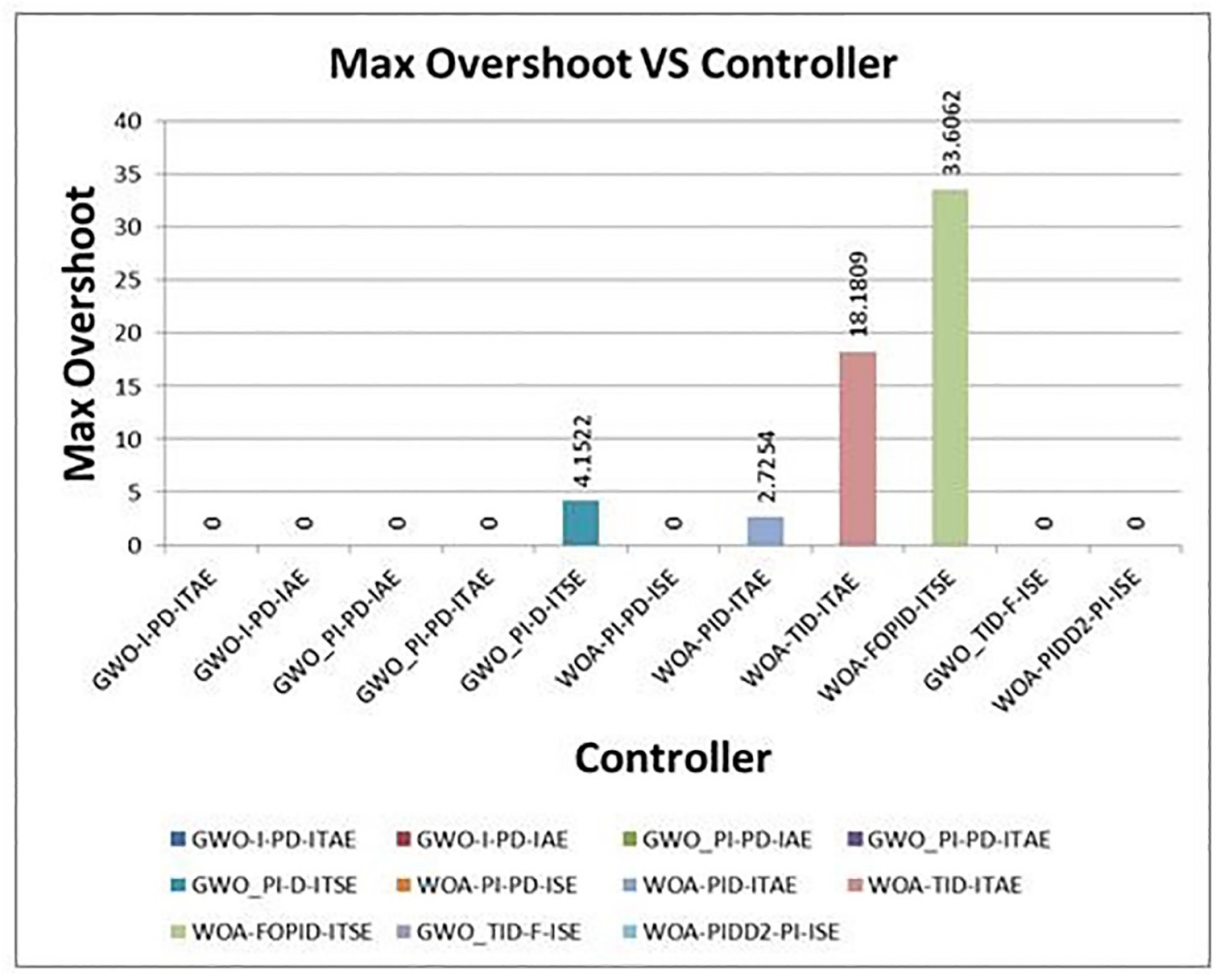
Graphical representation of Max overshoots vs. controllers.

Fig 12 shows the comparison of step response of optimum controllers with proposed controllers. The proposed controller results are excellent compare to others. The comparison of step response characteristics of proposed controllers with selected optimal controllers are shown in Table 7. The graphical representations of rise time, settling time and overshoot time vs different controllers are shown in Figs 13–15 respectively.

**Table 7:** Step response characteristics of WOA-PIDD^2^-PI.

Controller	Performance Parameters		
	Rise Time	Settling time	% Overshoot
**GWO-I-PD-ITAE**	0.5865	1.0080	0
**GWO-I-PD-IAE**	0.3669	0.6394	0
**GWO_PI-PD-IAE**	0.1777	1.1701	0
**GWO_PI-PD-ITAE**	0.2912	0.5449	0
**GWO_PI-D-ITSE**	0.1153	1.0444	4.1522
**WOA-PI-PD-ISE**	1.2080	2.1895	0
**WOA-PID-ITAE**	0.1244	0.3037	2.7254
**WOA-TID-ITAE**	0.9738	4.5982	18.1809
**WOA-FOPID-ITSE**	0.1252	1.1075	33.6062
**GWO_TID-F-ISE**	0.0059	0.0099	0
**WOA-PIDD^2^-PI-ISE**	0.0014	0.0025	0

### Case study

This section provides an in-depth analysis and comparison between different control schemes and optimization techniques to demonstrate the improved performance and robustness of the proposed control schemes. For a direct comparison with CSA-PI-PD [20] as case-01, the performance of the proposed control schemes GWO-TID-F-ISE and WOA-PIDD-PI-ISE can be evaluated using the BBS parameters given in Table 1. Table 8 represents the step response Comparison of proposed controllers with CSA-PI-PD [20].

**Table 8:** Step response comparison of proposed controllers with CSA-PI-PD [20].

Controller	Performance Parameters		
	Rise Time	Settling time	% Overshoot
**GWO-TID-F-ISE**	0.0059	0.0099	0
**WOA-PIDD^2^-PI-ISE**	0.0286	0.0521	0
**CSA-PI-PD- ITAE [20]**	0.77	1.21	0

We then consider BBS with change in its parameters as given in Eq 18, to test the robustness, step response performance, set point tracking and disturbance rejection capabilities of the proposed control schemes GWO-TID-F-ISE and WOA-PIDD-PI-IS, as well as provide the comparison with published relevant research work [21–24] as a case-02.

#### Case-01

In this section, a comparison is made between the proposed control strategies GWO-TID-F-ISE and WOA-PIDD^2^-PI-ISE with CSA-PI-PD [20] using the same model parameters given in Table 1 for BBS.

**Fig 16:**
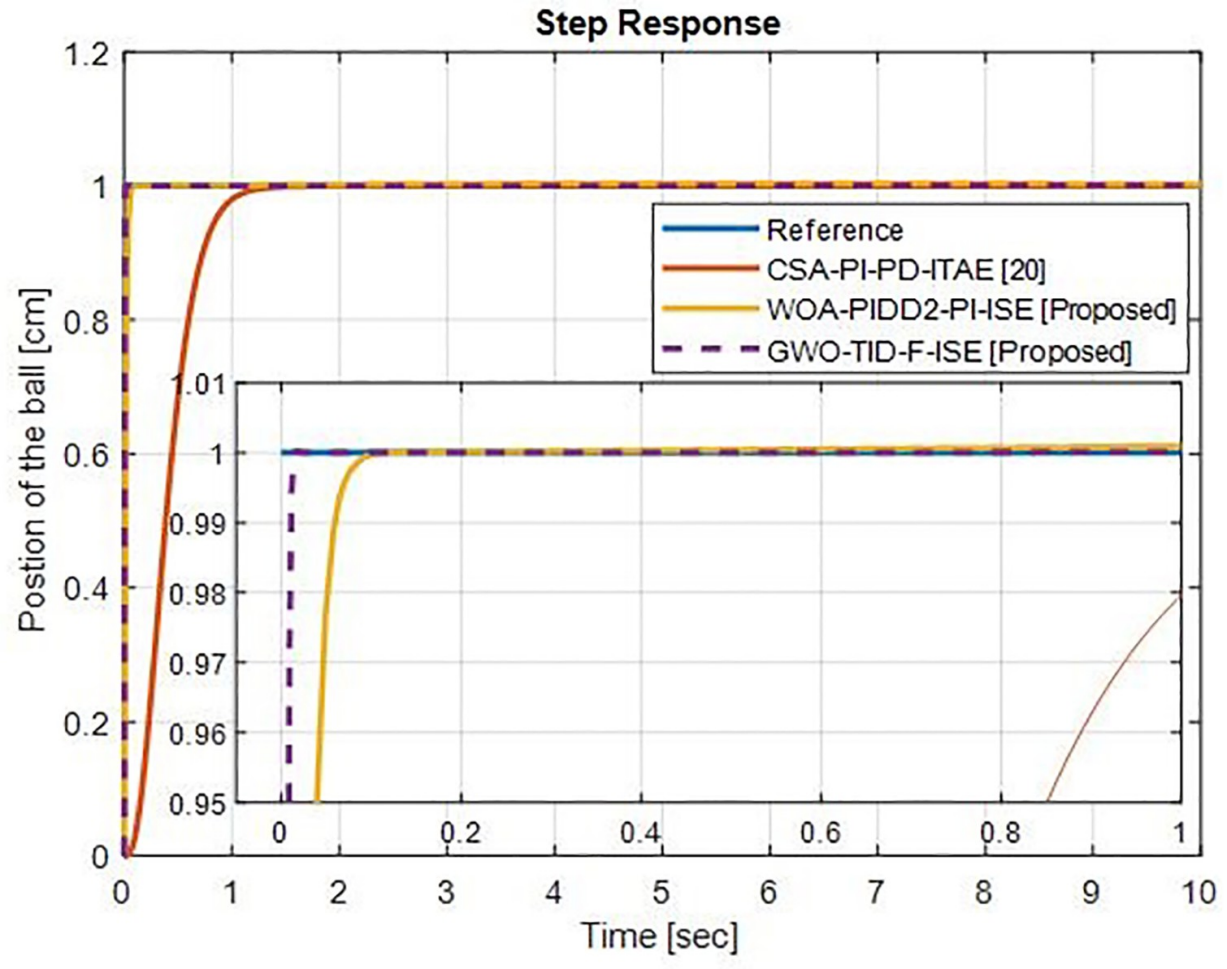
Step response Comparison of proposed controllers with CSA-PI-PD-ITAE.

In Fig 16 the proposed controllers are compared with CSA-PI_PD_ITAE. There is a significant improvement in rise time and settlement time for WOA-PIDD^2^-PI and GWO-TID-F-ISE over CSA-PI_PD-ITAE. The proposed controller WOA- PIDD^2^-PI-ISE reduce the rise time 96.28% and settling time is improve 95.69% then CSA-PI_PD-ITAE]. The proposed controller GWO-TID-F-ISE improve the rise time 99.33% and settling time is reduce 99.18% then CSA-PI-PD-ITAE.

#### Case-02

Here we consider the BBS with change in the parameters of the system as given in Eq 18 for a direct comparison with [21–24] and show the improved performance and robustness of the proposed schemes. A step response analysis, tracking performance, disturbance rejection analysis is carried out and comparison between different control schemes and optimization techniques are made.

The transfer function for the BBS [21–24] with the same values from Table 1 except the length of beam which is used as L = 0.4 m is given as

$$\begin{array}{c} G\left(s\right)=\frac{mgd}{L(\frac{J}{r^{2}}+m)}.\frac{1}{s^{2}}=\frac{0.7}{s^{2}}. \end{array}$$ (18)

In Table 9 Comparison of the proposed controller GWO-TID-F-ISE and WOA-PIDD^2^-PI-ISE step response performance with GA-PI-PD [21], SIMC-PID & H-Infinity [22], CDM-PID [23] and SA-PID & SA-PIDA [24] is presented.

**Table 9:** Step response comparison of proposed controllers with GA-PI-PD [21], SIMC-PID & H-Infinity [22], CDM-PID [23] and SA-PID & SA-PIDA [24].

Controller	Performance Parameters		
	Rise Time	Settling time	% Overshoot
**GWO-TID-F-ISE**	0.0094	0.0161	0
**WOA_PIDD^2^-PI-ISE**	0.0113	0.1338	0.3368
**GA-PI-PD- ITAE [21]**	0.70	1.08	1.00
**GA-PI-PD- ISE [21]**	0.46	3.24	0
**SIMC-PID [22]**	1.00	10.9	40
**H-Infinity [22]**	1.1	3.7	6.7
**SA-PID [24]**	0.4122	4.34	43
**SA-PIDA [24]**	0.381	3.94	38
**CDM-PID [23]**	0.3	1.4	0.5

**Fig 17:**
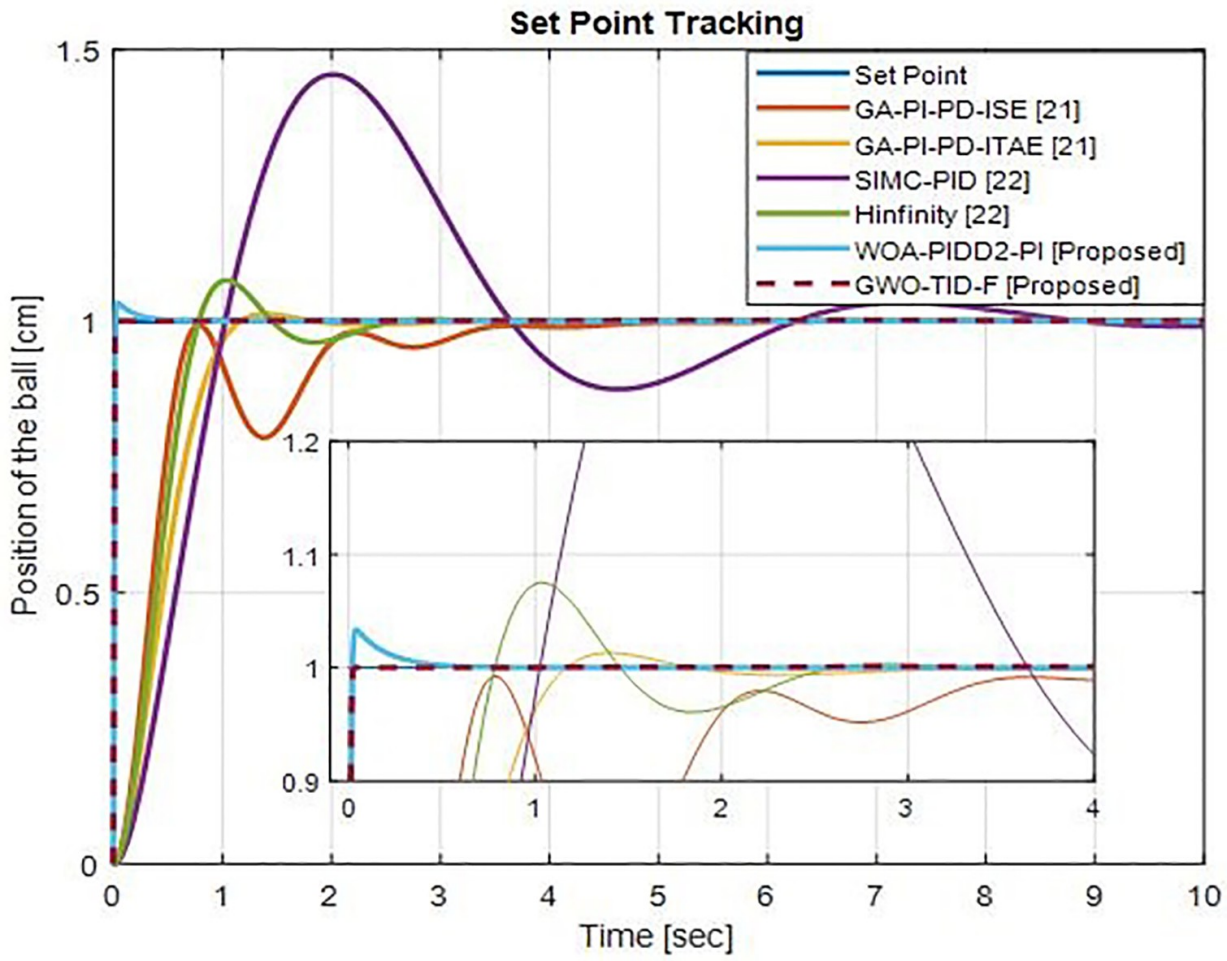
Step response of WOA-PIDD^2^-PI and GWO-TID-F controller vs. GA-PI-PD [21], SIMC-PID and H-infinity controller [22].

A comparison of the step response of the proposed controllers WOA-PIDD^2^-PI-ISE and GWO-TID-F-ISE with the GA-PI-PD, SIMC-PID and H-infinity controller is shown in Fig 17. As can be seen, the proposed controller WOA-PIDD^2^-PI-ISE rise in 0.0113 s and settled in 0.1338 s with a small overshoot. In the other proposal, GWO-TID-F-ISE rises within 0.0094 s and settles in 0.0161 s with no overshoot at all.

*(a) Set point tracking*. The author [21] discussed set point tracking comparison of their proposed controllers GA-PI-PD-ISE and GA-PI-PD-ITAE [21] with SIMC-PID and H-infinity [22]. So the suggested controller WOA-PIDD^2^-PI-ISE and GWO-TID-F-ISE are also compared with GA-PI-PD [21], SIMC-PID and H-infinity controller [22] by taking the same reference tracking signal.

**Fig 18:**
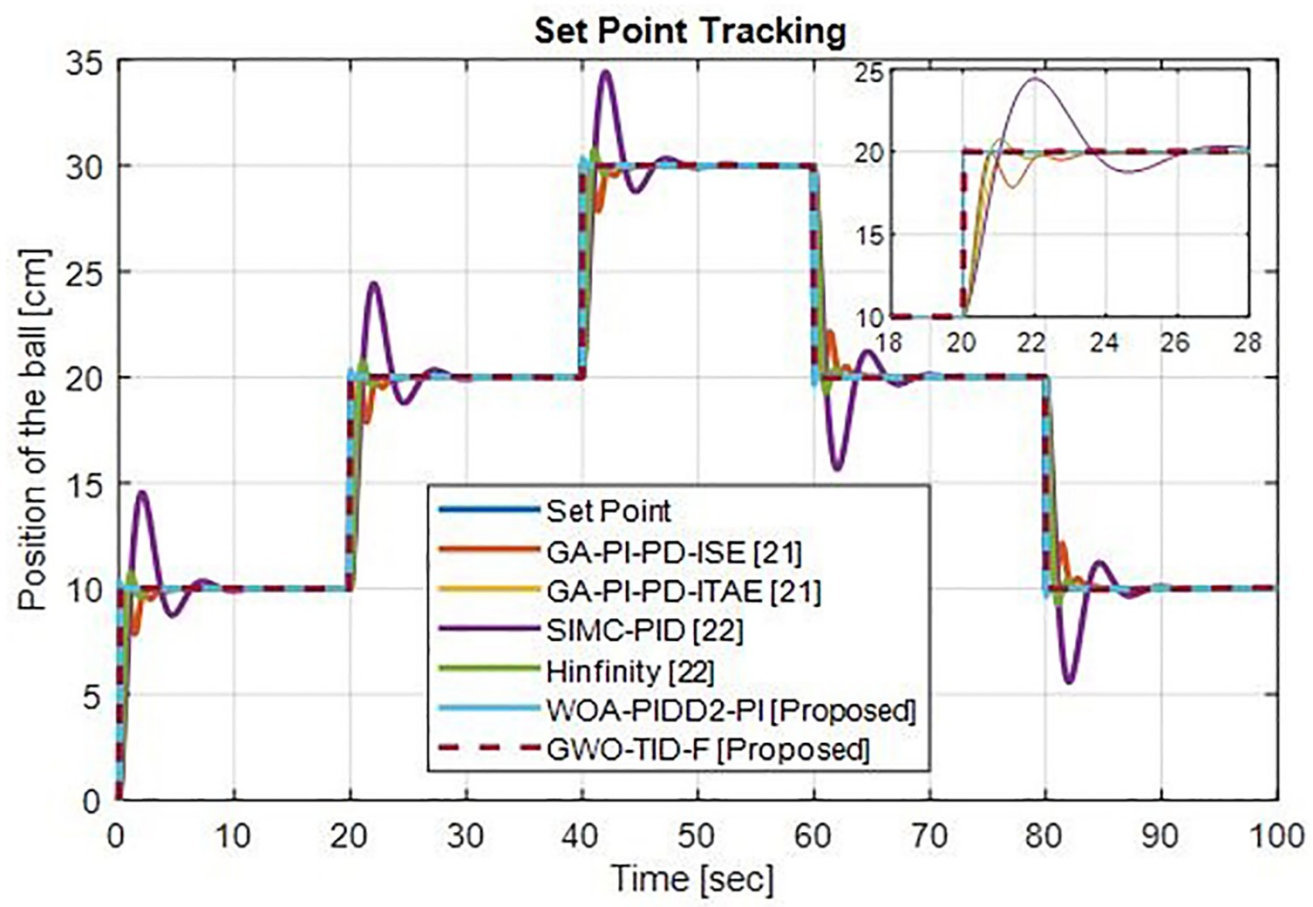
Set point tracking of WOA-PIDD^2^-PI-ISE and GWO-TID-F-ISE controller vs. GA-PI-PD, SIMC-PID and H-infinity controller.

Fig 18 shows the simulation results of WOA-PIDD^2^-PI-ISE and GWO-TID-F-ISE with the GA-PI-PD [21], SIMC-PID and H-infinity controller [22]. It is evident that the proposed WOA-PIDD^2^-PI-ISE and GWO-TID-F-ISE track the reference signal with superior accuracy, quickly, and without overshooting. It also shows that the proposed controller WOA-PIDD^2^-PI tracks very well with minimal overshoots but better than the other four controllers used in [21, 22], however, GWO-TID-F-ISE is ideally able to track the reference signal with zero overshoot.

**Fig 19:**
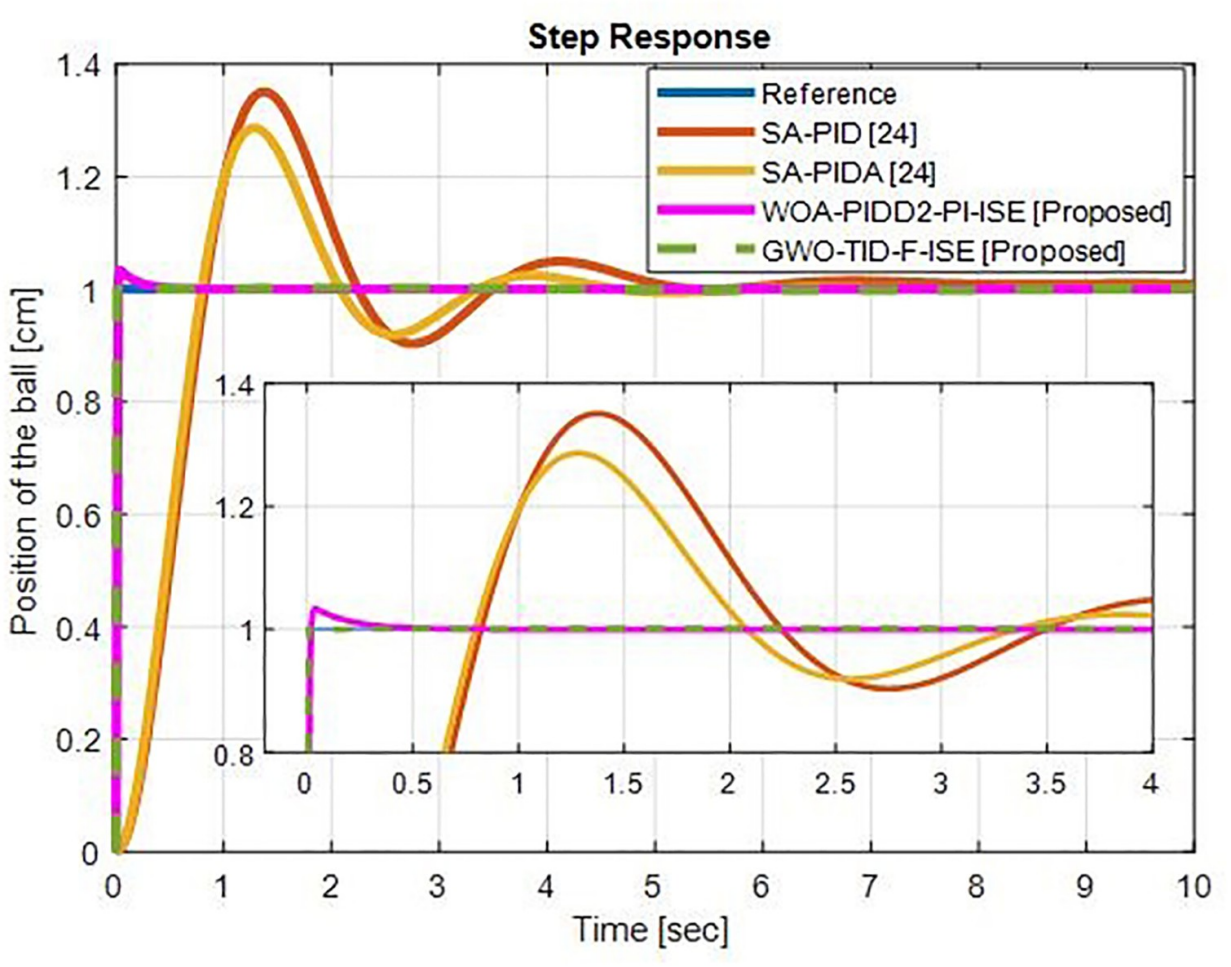
Step response of WOA-PIDD^2^-PI and GWO-TID-F controller vs. SA-PID & SA-PIDA controllers.

*(b) Step response comparison*. Fig 19 shows the step response comparison of proposed controllers with SA-PID & SA-PIDA [24]. The proposed controller WOA-PIDD^2^-PI reduces the rise time 97.25% and settling time 96.91% then SA-PID and decreases rise time 97.03% and Settling time 96.6%, then SA-PIDA. It also brings down overshoot effectively. The other proposed controller GWO-TID-F-ISE reduces rise time 97.71% and settling time 99. 62% compared to SA-PID and removes overshoot. It reduces rise time 97.53% and settling time 99.59% with zero overshoot than PIDA.

**Fig 20:**
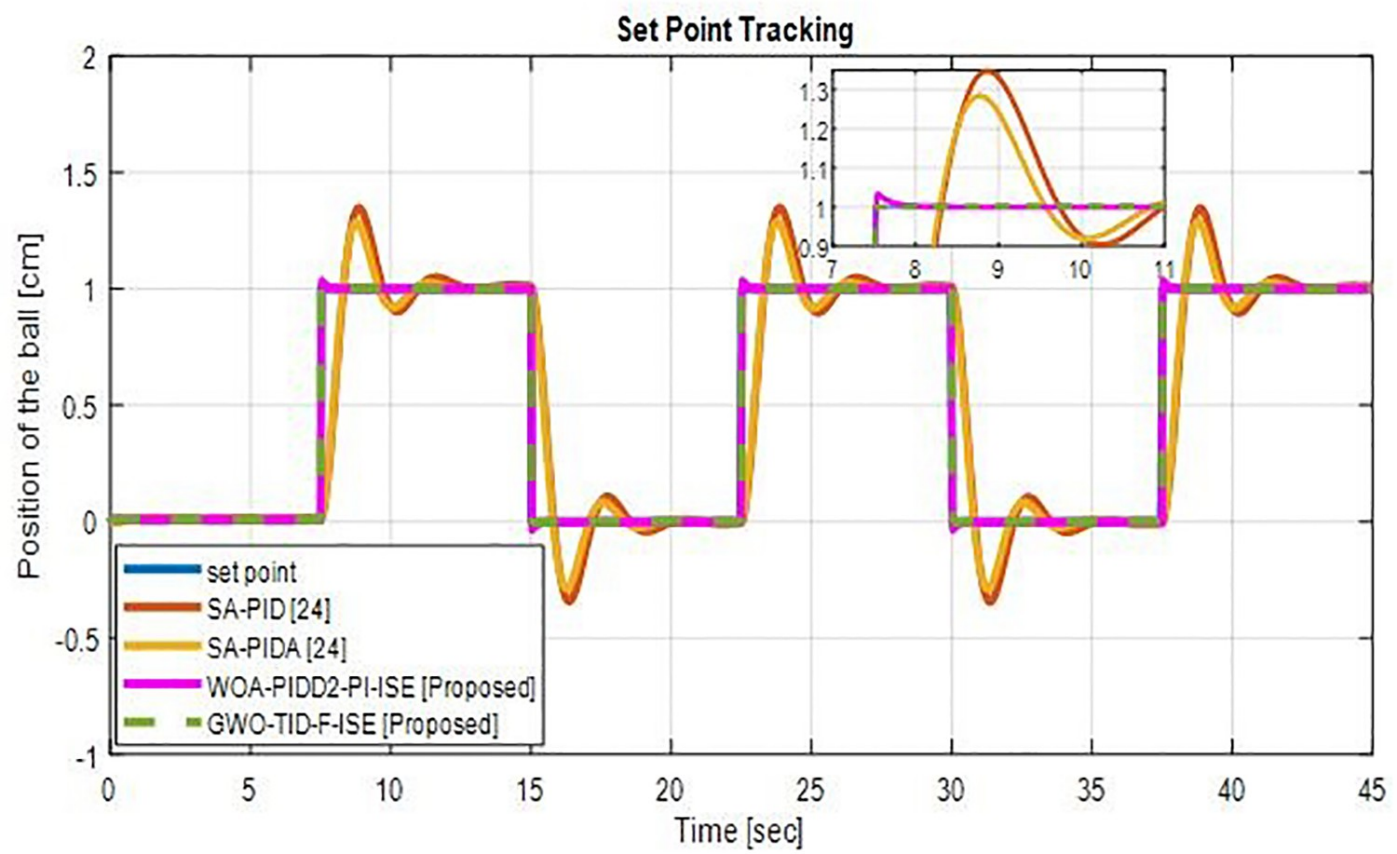
Set point tracking of WOA-PIDD^2^-PI and GWO-TID-F controller vs. SA-PID & SA-PIDA controllers.

*(c) Set point tracking*. Fig 20 shows the tracking comparison of proposed controllers with SA-PID and SA-PIDA [24]. WOA-PIDD^2^-PI-ISE has the ability to track the given signal quickly with minimal overshoot, while GWO-TID-F-ISE tracks signals ideally with no overshoot.

**Fig 21:**
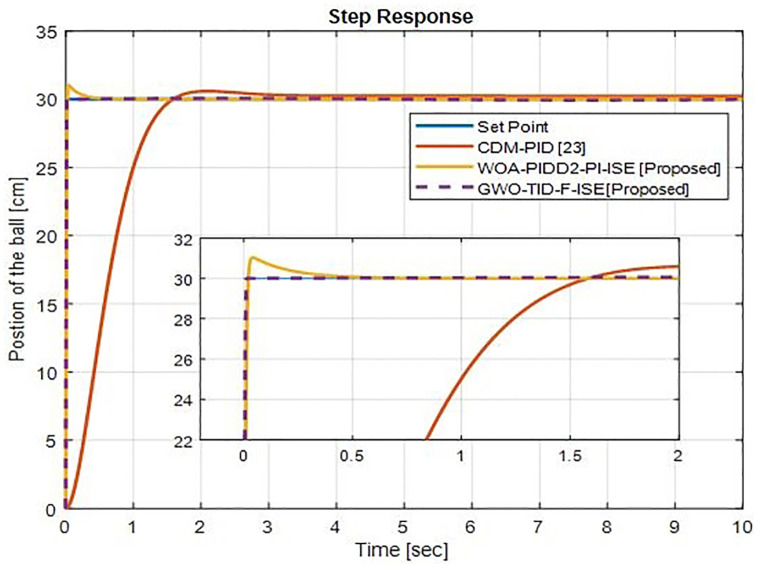
Comparison response with ball position at 30 cm input of WOA-PIDD^2^-PI-ISE and GWO-TID-F-ISE controller vs. CDM-PID.

*(d) Step response comparison*. The step response comparison between the proposed controllers and CDM-PID [23] is shown in Fig 21. The proposed controller, WOA-PIDD^2^-PI-ISE, significantly reduces rise time and settling time than CDM-PID with a small overshoot. The proposed controller GWO-TID-F-ISE drastically reduces the rise time and settling time then CDM-PID with zero overshoot.

**Fig 22:**
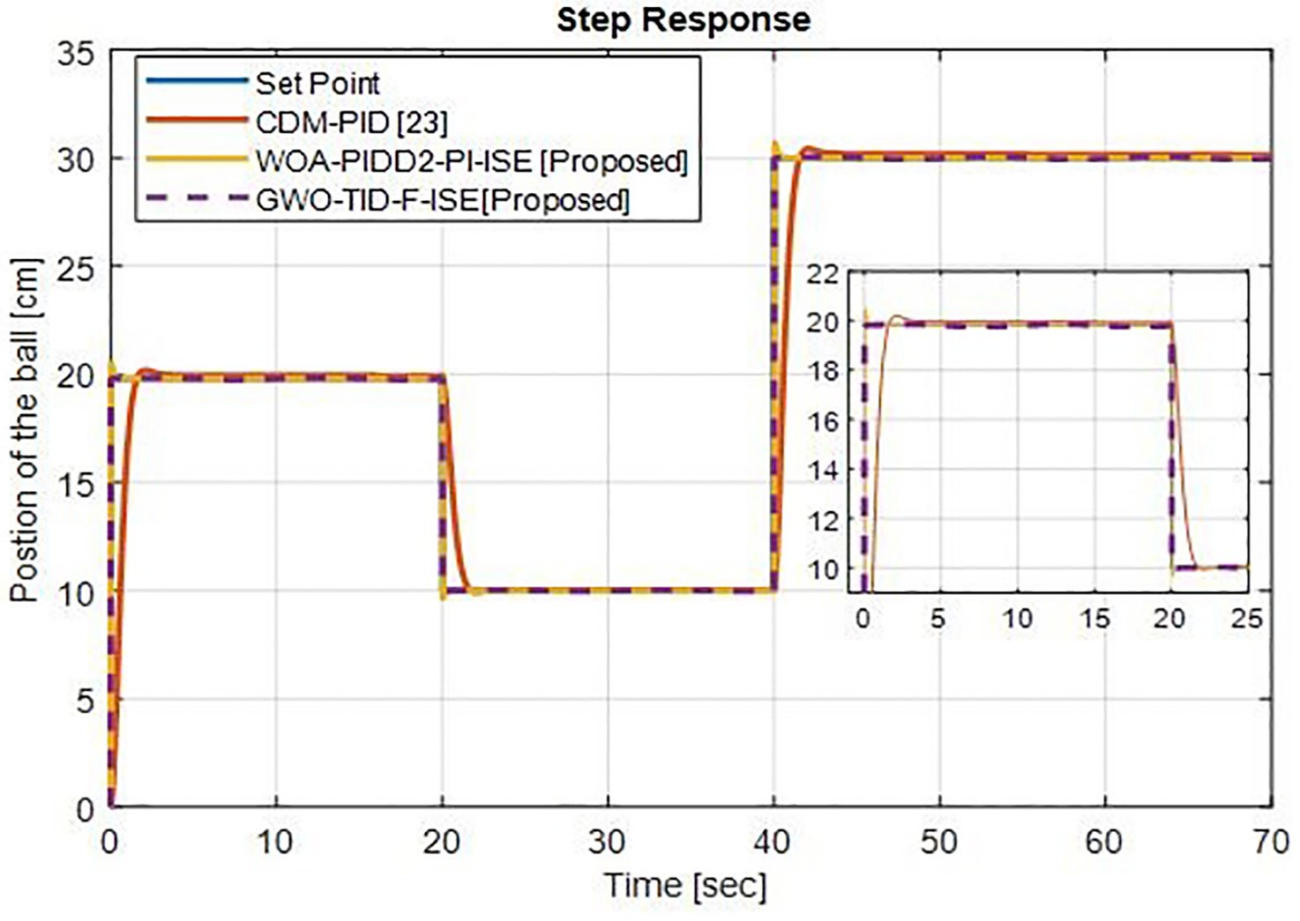
Response at different operating points of ball’s position (20 cm, 10 cm and 30 cm) of WOA-PIDD^2^-PI and GWO-TID-F controller vs. CDM-PID controller.

*(e) Set Point Tracking.* Fig 22 shows the tracking of different ball positions (20 cm, 10 cm and 30 cm) the proposed controller WOA-PIDD^2^-PI-ISE tracks very well with minimal overshoot but better than CDM-PID [23] controller and GWO-TID-F-ISE perfectly tracks the reference signal with zero overshoot.

**Fig 23:**
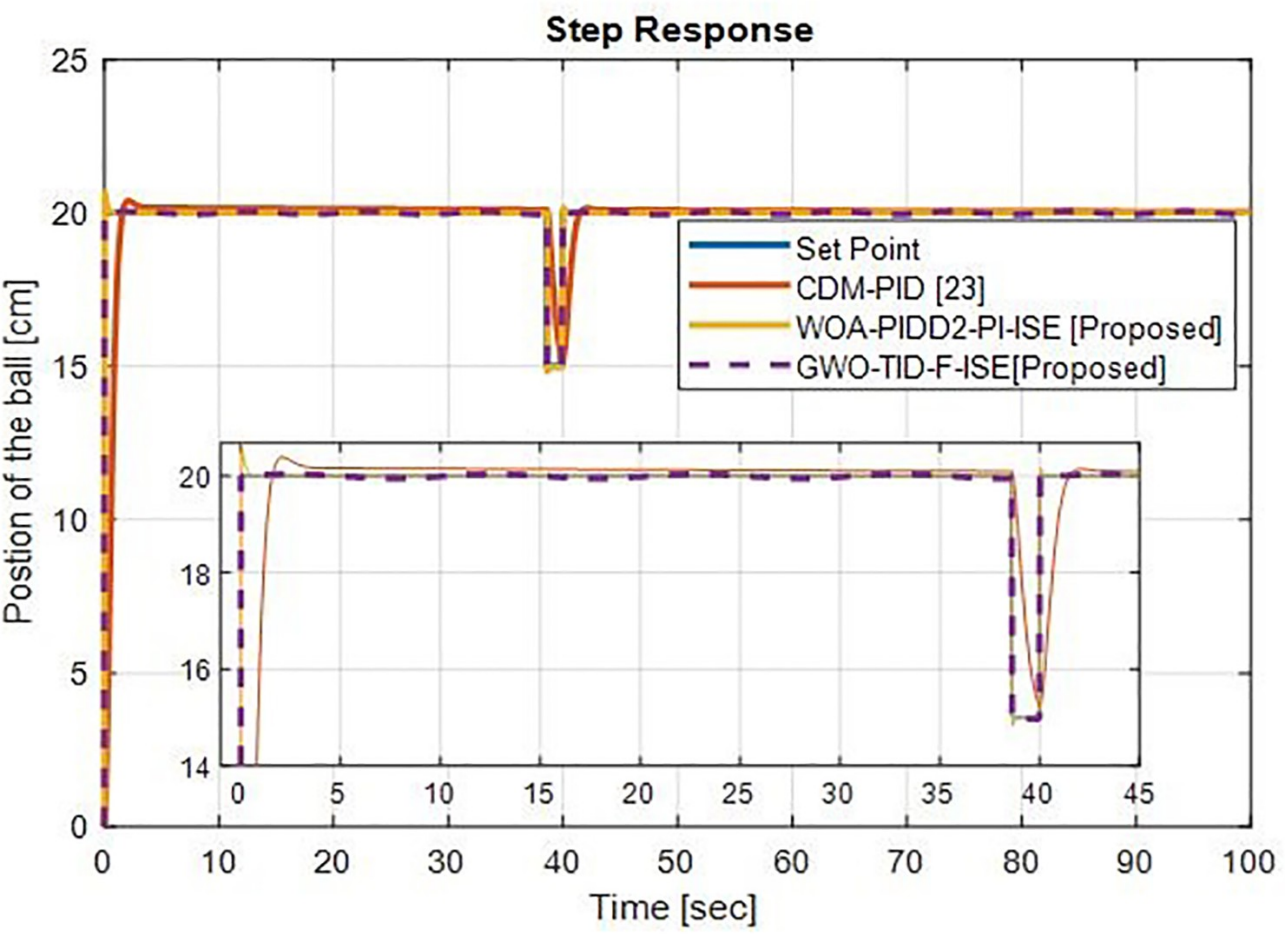
Disturbance rejection analyses at an operating point of 20cm of ball’s position of WOA-PIDD^2^-PI and GWO-TID-F controller vs. CDM-PID controller.

**Fig 24:**
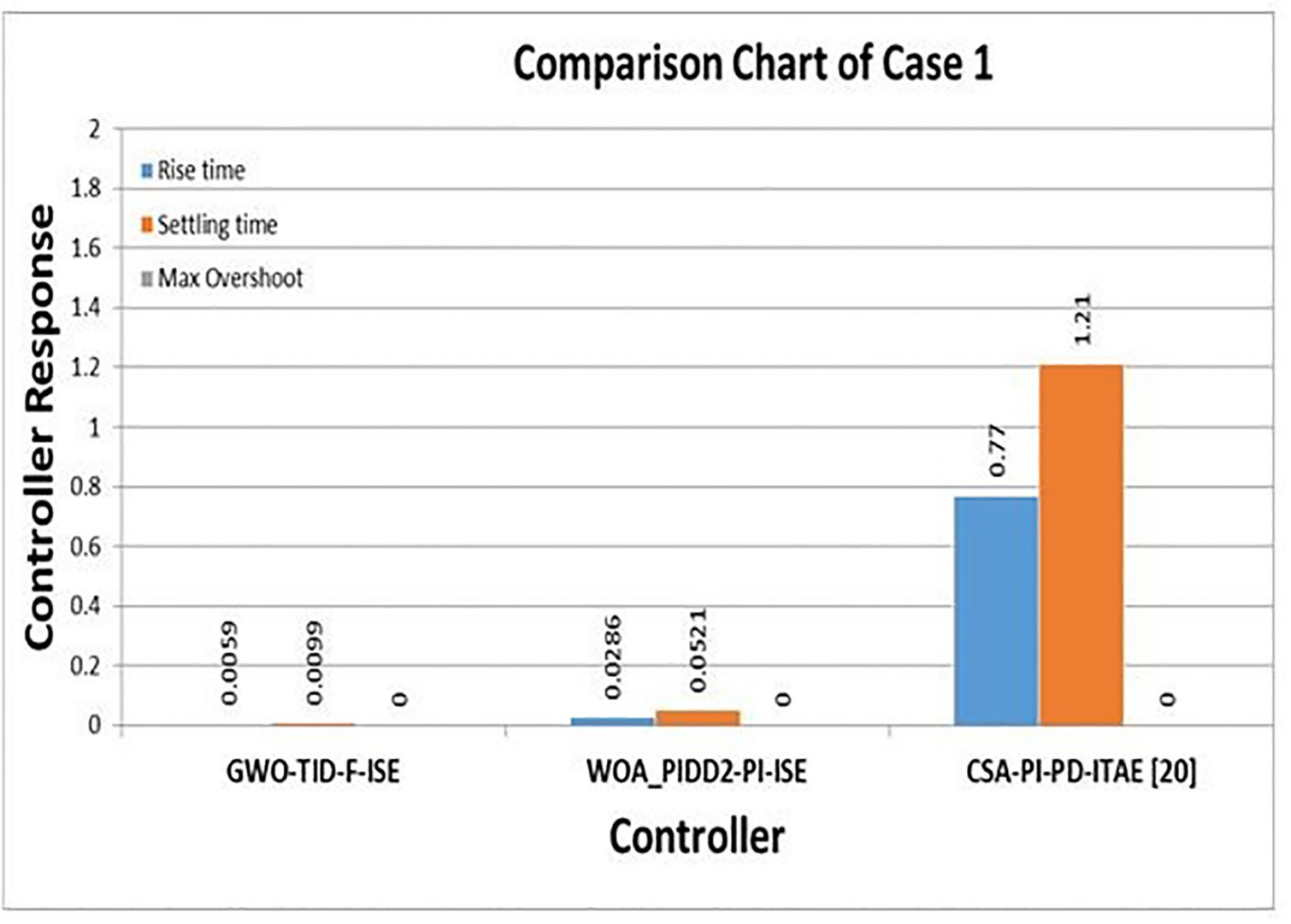
Graphical representation of WOA-PIDD^2^-PI and GWO-TID-F controller vs. CSA-PI-PD.

**Fig 25:**
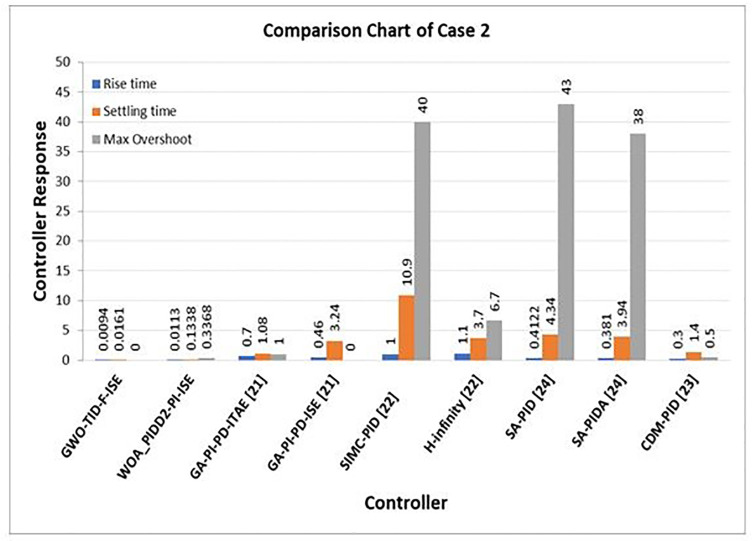
Graphical representation of WOA-PIDD^2^-PI and GWO-TID-F controller vs. GA-PI-PD [21], SIMC-PID & H-Infinity [22], CDM-PID [23] & SA-PID & SA-PIDA [24].

*(f) Step response comparison*. Here we consider reference signal subject to the disturbance as shown in Fig 23. The output response shows that the proposed controller WOA-PIDD^2^-PI-ISE is successful in disturbance rejection with minimal overshoot and much better than CDM-PID [23] controllers and GWO-TID-F-ISE is ideally successful in disturbance rejection with zero overshoot. The graphical representations of our proposed techniques vs different controllers are shown in Figs 24 and 25.

## Conclusion and future work

In this research, PIDD^2^-PI and Filtered TID (TID-F) controllers, optimized using grey wolf optimization (GWO) and whale optimization algorithm (WOA), are evaluated on the BBS to control the stability of the system with four performance indices ITAE, ITSE, ISE, and IAE. WOA-PIDD^2^-PI–ISE and GWO-TID-F-ISE give excellent performance. For additional validation, the proposed controllers are exclusively compared with TID, FOPID, I-PD, PI-D, and PI-PD, affirming that the proposed controllers exhibit superior performance regarding transient response. Furthermore, the robustness, set point tracking, and disturbance rejection of proposed controllers are compared with two case studies, and the findings are as follows:

The proposed controller WOA- PIDD^2^-PI-ISE reduced the rise time to 96.28% and the settling time to 95.69% than CSA-PI PD-ITAE [20]. The alternative proposed controller, GWO-TID-F-ISE, achieved a remarkable 99.33% reduction in rise time and a substantial 99.18% decrease in settling time compared to CSA-PI PD-ITAE [20]. WOA-PIDD^2^-PI-ISE effectively tracks the signal with minimal overshoot, surpassing the performance of GA-PI-PD-ISE and GA-PI-PD-ITAE [21], as well as SIMC-PID and H-infinity [22]. Meanwhile, GWO-TID-F-ISE demonstrates ideal signal tracking with zero overshoot, outperforming GA-PI-PD-ISE and GA-PI-PD-ITAE [21] and SIMC-PID and H-infinity [22]. WOA-PIDD^2^-PI-ISE reduced the rise time by 97.25%, and the settling time reduced by 96.91% than SA-PID [24] reduced the rise time by 97.03% and reduced the settling time by 96.6%, than SA-PIDA [24] it also minimizes overshoot greatly. GWO-TID-F-ISE reduces rise time by 97.71% and settling time by 99.62% compared to SA-PID [24] and removes overshoot. GWO-TID-F –ISE reduced rise time by 97.53% and settling time by 99.59% with zero overshoot than PIDA [24]. WOA-PIDD^2^-PI-ISE significantly reduces rise time and settling time compared to CDM-PID [23] with minimal overshoot. GWO-TID-F-ISE drastically reduces the rise time and settling time than CDM-PID with zero overshoot. WOA-PIDD^2^-PI-ISE exhibits excellent tracking performance with minimal overshoot, surpassing the capabilities of the CDM-PID controller [23]. On the other hand, GWO-TID-F-ISE perfectly tracks the reference signal with zero overshoot, indicating superior performance compared to both WOA-PIDD^2^-PI-ISE and the CDM-PID controller [23]. Moreover, WOA-PIDD^2^-PI-ISE excels in disturbance rejection with minimal overshoot, outperforming the CDM-PID controller [23], while GWO-TID-F-ISE achieves ideal disturbance rejection with zero overshoot.

Based on the simulation results, one can infer that GWO is more effective in tuning the proposed TID-F control than WOA. The TID-F works well with performance indexes such as ISE. Controller PIDD^2^-PI tuned best with WOA and performed excellently with performance index ISE. GWO-TID-F-ISE and WOA-PIDD^2^-PI-ISE are robust to control the stabilization and tracking performance of the underactuated BBS. In-depth analysis and comparison between different control schemes and optimization techniques confirm better dynamic and steady- state performance, tracking performance, disturbance rejection capability, and the robustness of the parameter variation of the BBS.

## Supporting information

S1 Text (TXT)

## Glossary

*α*_*L*_(*t*): Load gear
*β*: Angle of Beam
*θ*: Rotation angle with the wheel
*d*: Offset of the lever arm
*F*_*d*_: Translational force generated by gravity
*F*_*u*_: Force from the ball’s inertia
*J*: Moment of inertia
*L*: Length of crossbar
*m*: Ball’s mass
*r*: Ball’s Radius
*r*: Torque of the ball
ABC: Artificial Bee Colony technique
AFSMC: Model Reference Adaptive Control
BBS: Ball and Beam System
CDM: Coefficient Diagram Method
EA: Evolutionary Algorithm
FOPID: Fractional Order Proportional Integral Derivative
FSC: Fuzzy-Sliding-Control
GWO: Grey Wolf Optimization
I-PD: Integral- Proportional Derivative
IAE: Integral Absolute Error
ISE: Integral Square Error
ITAE: Integral Time Absolute Error
ITSE: Integral Time Square Error
LQR: Linear Quadratic Regulator
NN: Neural Network
PI-PD: Proportional Integral–Proportional Derivative
PID: Proportional Integral Derivative
PIDD^2^-PI: Proportional Integral Derivative-second Derivative with a Proportional Integrator
PSO: Particle Swarm Optimization
SA: Simulated Annealing
SMC: Sliding Mode Controller
TID-F: Tilt Integral Derivative with Filter
TID: Tilt-Integral-Derivative
TORA: Translational Oscillator with Rotational Actuator
UAS: Underactuated Systems
UMS: Underactuated Mechanical Systems
VTOL: Vertical Takeoff and Landing
WOA: Whale Optimization Algorithm

## References

[1] Daraz A, Malik SA, Haq IU, Khan KB, Laghari GF, Zafar F. Modified PID controller for automatic generation control of multi-source interconnected power system using fitness dependent optimizer algorithm. PloS one. 2020;15(11):e0242428. doi: 10.1371/journal.pone.0242428 33216787 PMC7678963

[2] Anantachaisilp P, Lin Z. Fractional Order PID Control of Rotor Suspension by Active Magnetic Bearings. Actuators. 2017;6(1):4. doi: 10.3390/act6010004

[3] Daraz A, Malik SA, Basit A, Aslam S, Zhang G. Modified FOPID Controller for Frequency Regulation of a Hybrid Interconnected System of Conventional and Renewable Energy Sources. Fractal and Fractional. 2023;7(1):89. doi: 10.3390/fractalfract7010089

[4] Tajjudin M, Johari SNH, Aziz SA, Adnan R. Minimum ISE Fractional-order PID (FOPID) Controller for Ball and Beam Mechanism. In: 2019 IEEE 10th Control and System Graduate Research Colloquium (ICSGRC). IEEE; 2019. p. 152–155.

[5] Daraz A, Malik SA, Mokhlis H, Haq IU, Zafar F, Mansor NN. Improved-Fitness Dependent Optimizer Based FOI-PD Controller for Automatic Generation Control of Multi-Source Interconnected Power System in Deregulated Environment. IEEE Access. 2020;8:197757–197775. doi: 10.1109/ACCESS.2020.3033983

[6] Mehedi IM, Al-Saggaf UM, Mansouri R, Bettayeb M. Two degrees of freedom fractional controller design: Application to the ball and beam system. Measurement. 2019;135(3):13–22. doi: 10.1016/j.measurement.2018.11.021

[7] Choudhary R, Rai JN, Arya Y. Cascade FOPI-FOPTID controller with energy storage devices for AGC performance advancement of electric power systems. Sustainable Energy Technologies and Assessments. 2022;53(2):102671. doi: 10.1016/j.seta.2022.102671

[8] Soni R, Sathans. Optimal control of a ball and beam system through LQR and LQG. In: 2018 2nd International Conference on Inventive Systems and Control (ICISC). IEEE; 2018. p. 179–184.

[9] Daraz A, Basit A, Zhang G. Performance analysis of PID controller and fuzzy logic controller for DC-DC boost converter. PloS one. 2023;18(10):e0281122. doi: 10.1371/journal.pone.0281122 37856453 PMC10586690

[10] Ahmadi Kamarposhti M, Shokouhandeh H, Alipur M, Colak I, Zare H, Eguchi K. Optimal Designing of Fuzzy-PID Controller in the Load-Frequency Control Loop of Hydro-Thermal Power System Connected to Wind Farm by HVDC Lines. IEEE Access. 2022;10:63812–63822. doi: 10.1109/ACCESS.2022.3183155

[11] Arya Y. ICA assisted FTIDN controller for AGC performance enrichment of interconnected reheat thermal power systems. Journal of Ambient Intelligence and Humanized Computing. 2023;14(3):1919–1935. doi: 10.1007/s12652-021-03403-6

[12] Hajipour S, Pourhashem H, Chegini SN, Bagheri A. Optimized neuro observer-based sliding mode control for a nonlinear system using fuzzy static sliding surface. Applied Soft Computing. 2022;124:108904. doi: 10.1016/j.asoc.2022.108904

[13] Srivastava V, Srivastava S. Hybrid optimization based PID control of ball and beam system. Journal of Intelligent & Fuzzy Systems. 2022;42(2):919–928. doi: 10.3233/JIFS-189760

[14] Wong WK, Ming CI. A Review on Metaheuristic Algorithms: Recent Trends, Benchmarking and Applications. In: 2019 7th International Conference on Smart Computing & Communications (ICSCC). IEEE; 2019. p. 1–5.

[15] Gutierrez MK, Choi DM, Jula H. Using Genetic Algorithms to Optimize Control of a Ball-and-Beam System. In: 2020 IEEE Green Energy and Smart Systems Conference (IGESSC). IEEE; 2020. p. 1–6.

[16] Katoch S, Chauhan SS, Kumar V. A review on genetic algorithm: past, present, and future. Multimedia tools and applications. 2021;80(5):8091–8126. doi: 10.1007/s11042-020-10139-6 33162782 PMC7599983

[17] Jiang Y, Li J, Lv Y, Wang R. Adaptive Control of Ball and Beam System Using Knowledge-Based Particle Swarm Optimization. In: 2021 7th International Conference on Automation, Robotics and Applications (ICARA). IEEE; 2021. p. 168–172.

[18] Latif S, Irshad S, Ahmadi Kamarposhti M, Shokouhandeh H, Colak I, Eguchi K. Intelligent Design of Multi-Machine Power System Stabilizers (PSSs) Using Improved Particle Swarm Optimization. Electronics. 2022;11(6):946. doi: 10.3390/electronics11060946

[19] Ahmadi Kamarposhti M. Optimal control of islanded micro grid using particle swarm optimization algorithm. International Journal of Industrial Electronics Control and Optimization. 2018;1(1):53–60.

[20] Ali ET, Abdullah S, Amir M, Adeel EM. Stability control of ball and beam system using heuristic computation based PI-D and PI-PD controller. Technical Journal. 2019;24(01):21–29.

[21] Ali T, Malik SA, Adeel M, Amir M. Set point tracking of Ball and Beam System Using Genetic Algorithm based PI-PD Controller. NUST Journal of Engineering Sciences. 2018;11(1):12–16. doi: 10.24949/njes.v11i1.287

[22] Sathiyavathi S, Krishnamurthy K. PID control of ball and beam system–A real time experimentation. 2013;.

[23] Meenakshipriya B, Kalpana K. Modelling and Control of Ball and Beam System using Coefficient Diagram Method (CDM) based PID controller. IFAC Proceedings Volumes. 2014;47(1):620–626. doi: 10.3182/20140313-3-IN-3024.00079

[24] Sehgal K, Harsh. Modelling and Control of Dynamical Ball and Beam System Using SA Tuned PIDA and PIaD Controllers. In: 2021 IEEE International Conference on Electronics, Computing and Communication Technologies (CONECCT). IEEE; 2021. p. 1–6.

[25] Mishra D, Nayak PC, Prusty RC. PSO optimized PIDF controller for Load-frequency control of A.C Multi-Islanded-Micro grid system. In: 2020 International Conference on Renewable Energy Integration into Smart Grids: A Multidisciplinary Approach to Technology Modelling and Simulation (ICREISG). IEEE; 2020. p. 116–121.

[26] Dorigo M, Birattari M, Stutzle T. Ant colony optimization. IEEE Computational Intelligence Magazine. 2006;1(4):28–39. doi: 10.1109/CI-M.2006.248054

[27] Shokouhandeh H, Latif S, Irshad S, Ahmadi Kamarposhti M, Colak I, Eguchi K. Optimal Management of Reactive Power Considering Voltage and Location of Control Devices Using Artificial Bee Algorithm. Applied Sciences. 2022;12(1):27. doi: 10.3390/app12010027

[28] Singh N, Singh SB. Hybrid Algorithm of Particle Swarm Optimization and Grey Wolf Optimizer for Improving Convergence Performance. Journal of Applied Mathematics. 2017;2017(1–4):1–15. doi: 10.1155/2017/2030489

[29] Cazzolato BS, Prime Z. On the Dynamics of the Furuta Pendulum. Journal of Control Science and Engineering. 2011;2011(6):1–8. doi: 10.1155/2011/528341

[30] Cholodowicz E, Orlowski P. Furuta Pendulum Real-Time System with Brushless Dc Motor and Cascade Hybrid Control. In: 2020 16th International Conference on Control, Automation, Robotics and Vision (ICARCV). IEEE; 2020. p. 1123–1130.

[31] Moreno-Valenzuela J, Aguilar-Avelar C. Motion control of underactuated mechanical systems. vol. 1. Springer; 2018.

[32] Parulski P, Bartkowiak P, Pazderski D. Evaluation of Linearization Methods for Control of the Pendubot. Applied Sciences. 2021;11(16):7615. doi: 10.3390/app11167615

[33] Wang Y, Mao W, Xin B, Wang Q, Wei J. Cooperative Control of Rotating Inverted Pendulum Based on Fuzzy Control. In: 10th Int. Symp. on Computational Intelligence and Industrial Applications (ISCIIA 2022), Article. A5-1; 2022.

[34] Liu J, Zhuan X, Lu C. Swing-Up and Balance Control of Cart-Pole Based on Reinforcement Learning DDPG. In: Pan L, Zhao D, Li L, Lin J, editors. Bio-Inspired Computing: Theories and Applications. vol. 1801 of Communications in Computer and Information Science. Singapore: Springer Nature Singapore; 2023. p. 419–429.

[35] Gembalczyk G, Domogała P, Leśniowski K. Modeling of Underactuated Ball and Beam System—A Comparative Study. Actuators. 2023;12(2):59. doi: 10.3390/act12020059

[36] Ali HI, Jassim HM, Hasan AF. Optimal Nonlinear Model Reference Controller Design for Ball and Plate System. Arabian Journal for Science and Engineering. 2019;44(8):6757–6768. doi: 10.1007/s13369-018-3616-1

[37] Feng Q, Zhang A, Zhang X, Pang G, Liu Z. Robust Stabilization of Underactuated TORA System Based on Disturbance Observer and Fixed-Time Sliding Mode Control Method. Actuators. 2022;11(10):271. doi: 10.3390/act11100271

[38] Anand S, Prasad R. Dynamics and control of ball and beam system. Int J Recent Innov Trends Comput Commun. 2017;5(5):1332–1339.

[39] Versloot J, Parrott E, Dubay R. Adaptive Control of a Ball and Beam System. In: 2020 IEEE International Systems Conference (SysCon). IEEE; 2020. p. 1–7.

[40] Srivastava A, Pratap B. Nonlinear observer-based robust controller design for ball and beam system: an LMI-based approach. International Journal of Nonlinear Dynamics and Control. 2018;1(2):211. doi: 10.1504/IJNDC.2018.093629

[41] Ding M, Liu B, Wang L. Position control for ball and beam system based on active disturbance rejection control. Systems Science & Control Engineering. 2019;7(1):97–108. doi: 10.1080/21642583.2019.1575297

[42] Ravichandran P, Sathiyavathi S, Sathish Babu S, Vimala Starbino A. Hybrid Arrangement of Iterative Learning Control Strategy for Ball and Beam System. IETE Journal of Research. 2023;69(2):916–923. doi: 10.1080/03772063.2020.1844072

[43] Shirke H, Kulkarni N. Mathematical modeling, simulation and control of ball and beam system. International Journal of Engineering Research & Technology. 2015;4(3):834–838.

[44] Bolívar-Vincenty CG, Beauchamp-Báez G. Modelling the ball-and-beam system from newtonian mechanics and from lagrange methods. In: Twelfth LACCEI Latin American and Caribbean Conference for Engineering and Technology. vol. 22; 2014. p. 24.

[45] Singh K, Amir M, Arya Y. Optimal dynamic frequency regulation of renewable energy based hybrid power system utilizing a novel TDF-TIDF controller. Energy Sources, Part A: Recovery, Utilization, and Environmental Effects. 2022;44(4):10733–10754. doi: 10.1080/15567036.2022.2158251

[46] Rajput GK, Yadav A, Kumar A, Gautam A, Tiwari A, Babu NR, et al. Design of TID controller based on firefly algorithm for controlling the speed of a D.C. Motor. E3S Web of Conferences. 2020;184:01038. doi: 10.1051/e3sconf/202018401038

[47] Dada E, Joseph S, Oyewola D, Fadele AA, Chiroma H, Abdulhamid SM. Application of Grey Wolf Optimization Algorithm: Recent Trends, Issues, and Possible Horizons. Gazi University Journal of Science. 2022;35(2):485–504. doi: 10.35378/gujs.820885

[48] Sharma I, Kumar V, Sharma S. A Comprehensive Survey on Grey Wolf Optimization. Recent Advances in Computer Science and Communications. 2022;15(3):1.

[49] Hou Y, Gao H, Wang Z, Du C. Improved Grey Wolf Optimization Algorithm and Application. Sensors (Basel, Switzerland). 2022;22(10). doi: 10.3390/s22103810 35632219 PMC9147573

[50] Şen MA, Kalyoncu M. Grey wolf optimizer based tuning of a hybrid LQR-PID controller for foot trajectory control of a quadruped robot. Gazi University Journal of Science. 2019;32(2):674–684.

[51] Precup RE, Voisan EI, Petriu EM, Tomescu ML, David RC, Szedlak-Stinean AI, et al. Grey Wolf Optimizer-Based Approaches to Path Planning and Fuzzy Logic-based Tracking Control for Mobile Robots. International journal of computers communication & Control. 2020;15(3).

[52] Djerioui A, Houari A, Machmoum M, Ghanes M. Grey Wolf Optimizer-Based Predictive Torque Control for Electric Buses Applications. Energies. 2020;13(19):5013. doi: 10.3390/en13195013

[53] Nayak PC, Rath S, Prusty RC. Performance Analysis of different FACTS devices using Grey Wolf Optimization algorithm PDF plus (1+PI) controller based multi-area AGC system. In: 2020 International Conference on Renewable Energy Integration into Smart Grids: A Multidisciplinary Approach to Technology Modelling and Simulation (ICREISG). IEEE; 2020. p. 143–148.

[54] Hekimoglu B, Ekinci S. Optimally Designed PID Controller for a DC-DC Buck Converter via a Hybrid Whale Optimization Algorithm with Simulated Annealing. Electrica. 2020;20(1):19–27. doi: 10.5152/electrica.2020.19034

[55] Kumar R, Singh R, Ashfaq H, Singh SK, Badoni M. Power system stability enhancement by damping and control of Sub-synchronous torsional oscillations using Whale optimization algorithm based Type-2 wind turbines. ISA transactions. 2021;108:240–256. doi: 10.1016/j.isatra.2020.08.037 32888728

[56] Loucif F, Kechida S, Sebbagh A. Whale optimizer algorithm to tune PID controller for the trajectory tracking control of robot manipulator. Journal of the Brazilian Society of Mechanical Sciences and Engineering. 2020;42(1):1280. doi: 10.1007/s40430-019-2074-3

[57] Mirjalili S, Lewis A. The Whale Optimization Algorithm. Advances in Engineering Software. 2016;95(12):51–67. doi: 10.1016/j.advengsoft.2016.01.008

[58] Han Q, Yang X, Song H, Sui S, Zhang H, Yang Z. Whale Optimization Algorithm for Ship Path Optimization in Large-Scale Complex Marine Environment. IEEE Access. 2020;8:57168–57179. doi: 10.1109/ACCESS.2020.2982617

